# Visualizing the native cellular organization by coupling cryofixation with expansion microscopy (Cryo-ExM)

**DOI:** 10.1038/s41592-021-01356-4

**Published:** 2022-01-13

**Authors:** Marine H. Laporte, Nikolai Klena, Virginie Hamel, Paul Guichard

**Affiliations:** Department of Cell Biology, University of Geneva, Geneva, Switzerland

**Keywords:** Fluorescence imaging, Super-resolution microscopy

## Abstract

Cryofixation has proven to be the gold standard for efficient preservation of native cell ultrastructure compared to chemical fixation, but this approach is not widely used in fluorescence microscopy owing to implementation challenges. Here, we develop Cryo-ExM, a method that preserves native cellular organization by coupling cryofixation with expansion microscopy. This method bypasses artifacts associated with chemical fixation and its simplicity will contribute to its widespread use in super-resolution microscopy.

## Abstract (web summary)

Cryo-ExM combines the expansion microscopy for super-resolution imaging with cryofixation for ultrastructure preservation. Cryo-ExM outperforms established fixation methods on a range of sensitive subcellular structures.

## Main

In super-resolution fluorescence microscopy (SRM), which now encompasses expansion microscopy (ExM)^1^, it is possible to locate proteins with nanometer resolution in a cellular context^2^. However, SRM often requires cell fixation with aldehyde-based chemical crosslinkers, such as paraformaldehyde or protein precipitation with cold methanol, both followed by cell permeabilization, all of which potentially alter the native cellular state and indirectly the interpretations that follow^3^. This issue was raised decades ago in electron microscopy (EM) where years of development resulted in demonstrating that cryofixation (rapidly fixing the cell in a vitreous state), is the sole approach for preserving the native ultrastructure^4,5^.

To address this important issue, several groups developed SRM on cryofixed samples, such as cryo-SIM^6,7^, cryo-SMLM^8^ or cryo-SOFI^9^, but these approaches often require sophisticated custom setups, highly stable cryostages, as well as specific high NA long-working-distance air objectives to avoid sample devitrification, limiting their use by most laboratories.

On the other hand, applying our optimized expansion microscopy protocol U-ExM^10^ on unfixed cells preserves the ultrastructure of the centriole, a stable microtubule-based organelle; however, we also observed that the complete lack of fixation in living cells leads to the loss of some cellular elements such as cytoplasmic microtubules^11^. We therefore were motivated to develop Cryo-ExM, a method that combines the advantages of expansion microscopy for super-resolution imaging and cryopreservation.

## Results

### Combining cryofixation with expansion microscopy

**Fig. 1:**
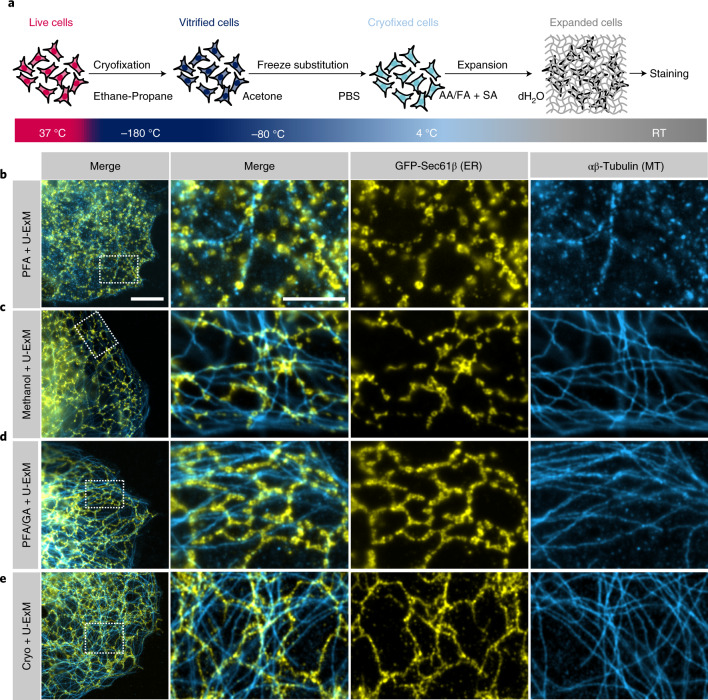
Cell architecture preservation in cryo versus chemical fixations. **a**, Cryo-ExM protocol pipeline. AA/FA, acrylamide/formaldehyde, SA, sodium acrylate, RT, room temperature. **b**–**e**, Widefield image of PFA-fixed (**b**), methanol-fixed (**c**), PFA/GA-fixed (**d**) or cryofixed (**e**) U2OS cells processed for U-ExM. Cells expressing the ER marker GFP-Sec61β are stained with α/β-tubulin (cyan) and GFP (yellow). White dotted squares correspond to the adjacent insets. U-ExM, ultrastructure expansion microscopy. Scale bars, 5 μm and 2 μm (insets).

**Extended Data Fig. 1:**
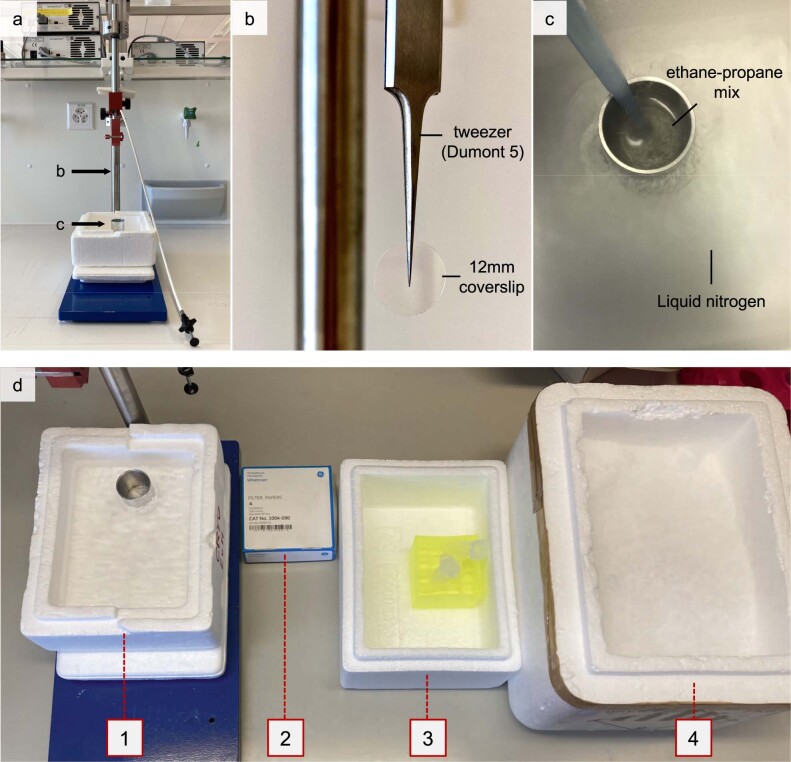
Cryo-plunging set up. (**a**) Manual plunge-freezing system. The tweezer holding the coverslip is placed as shown in (**b**) in the plunger arm above a metal cylinder chilled in liquid nitrogen and filled with ethane-propane mix (**c**). (**d**) Once the system ready (1), held coverslips are carefully blotted (2), plunged, transferred into eppendorf containing acetone chilled with liquid nitrogen (3) and incubated overnight in dry ice (4).

To develop Cryo-ExM, we capitalized on the well-known EM approach of cryofixation followed by freeze substitution, which consists of embedding a vitrified specimen in a resin before polymerization and subsequently processing for EM studies. This approach allows for high levels of cellular architecture preservation and far surpasses chemical fixation^8,12^. We reasoned that EM resin could be replaced by the expansion microscopy hydrogel (Fig. 1a). To do so, biological samples grown on glass coverslips are first cryofixed using conventional rapid plunging in liquid ethane–propane to form vitreous ice, as used in cryo-FIB-milling studies^13^ (Extended Data Fig. 1). Then, vitrified coverslips are incubated in acetone pre-cooled with liquid nitrogen (−180 °C) and placed on dry ice (−80 °C) overnight, allowing dry ice evaporation and a gradual rise in sample temperature from −180 °C to 0 °C. During this step, the water of the sample is slowly replaced by acetone, a crucial process ensuring proper cell architecture preservation^14^. Samples are next rehydrated with sequential baths of ethanol mixed with an increasing amount of water and proceed for ExM hydrogel embedding using the U-ExM protocol^10^ (Methods).

### Cryofixation surpasses chemical fixations when coupled to U-ExM

**Extended Data Fig. 2:**
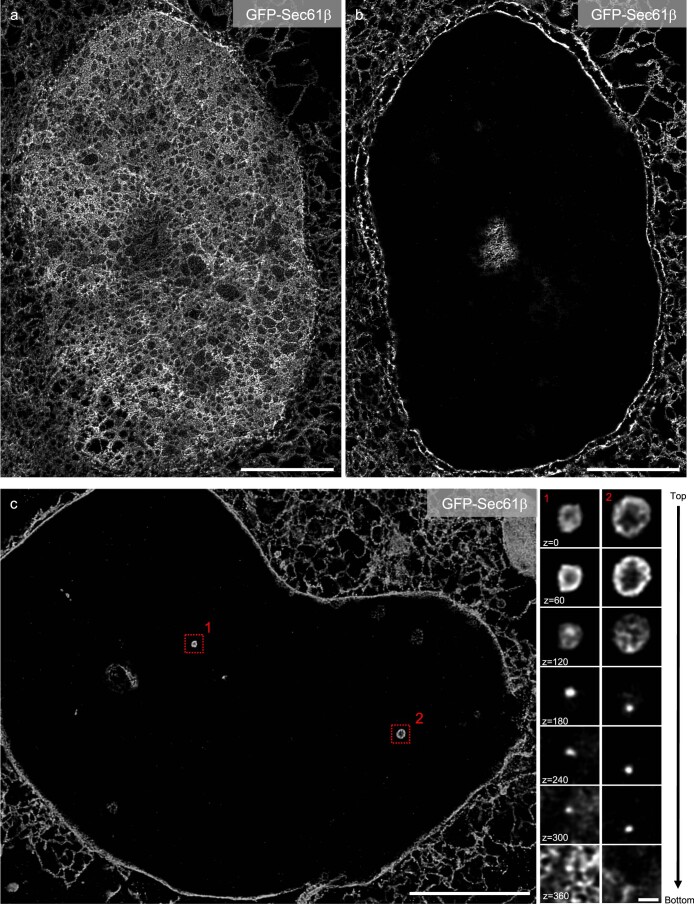
Nuclear-associated reticulum observed by cryo-ExM. (**a**-**c**) Confocal images of cryo-fixed U2OS cell expressing the ER marker GFP-Sec61β, expanded and stained for GFP (grey). Single plans at different Z-positions show the preservation of the reticulum surrounding the nucleus from the top (**a**) and the side (**b**, **c**) of the nucleus. (**c**). Z-section across the nucleus showing the presence of nucleoplasmic reticulum invaginating inside the nucleus (red dashed squares). Insets show the invaginations inside the nucleus through the z-axis (1, 2). Scale bars = 5 μm (**a**-**c**) and 200 nm (insets 1, 2).

To validate Cryo-ExM, we first chose to simultaneously visualize a membrane-based organelle, the endoplasmic reticulum (ER) using GFP-Sec61β as a proxy^8^ and the microtubule (MT) cytoskeleton using α/β-tubulin antibodies, two dynamic subcellular structures that are sensitive to chemical fixation^8,11^ (Fig. 1). We compared expanded U2OS cells treated with aldehydes (paraformaldehyde (PFA) alone and a mix of PFA and glutaraldehyde (GA)), methanol or cryofixed (Fig. 1b–e). As previously observed^8^, we found that PFA fixation disrupted both ER and microtubule integrity, as these structures seemed fragmented (Fig. 1b). Similarly, both methanol and PFA/GA fixations affected ER morphology, though to a lesser extent, giving rise to a less-fragmented ER, while preserving microtubules (Fig. 1c,d). In contrast, Cryo-ExM led to thin nonfragmented ER tubules, which is in agreement with live SRM data^15^. Overall, Cryo-ExM provided good preservation of the cellular organization with microtubules that seemed fully intact (Fig. 1e), demonstrating that cryofixation, freeze substitution and expansion microscopy procedures are compatible with ER and microtubule ultrastructure organization. Moreover, we also noticed that this approach preserves the cellular organization deep inside the cell allowing visualization of the perinuclear reticulum as well as the so-called nucleoplasmic reticulum, which corresponds to dynamic convolutions of the nuclear envelope, including deep tubular invaginations of variable length, from ~200 nm diameter to start, narrowing toward the inside the nucleus^16^ (Extended Data Fig. 2).

**Fig. 2:**
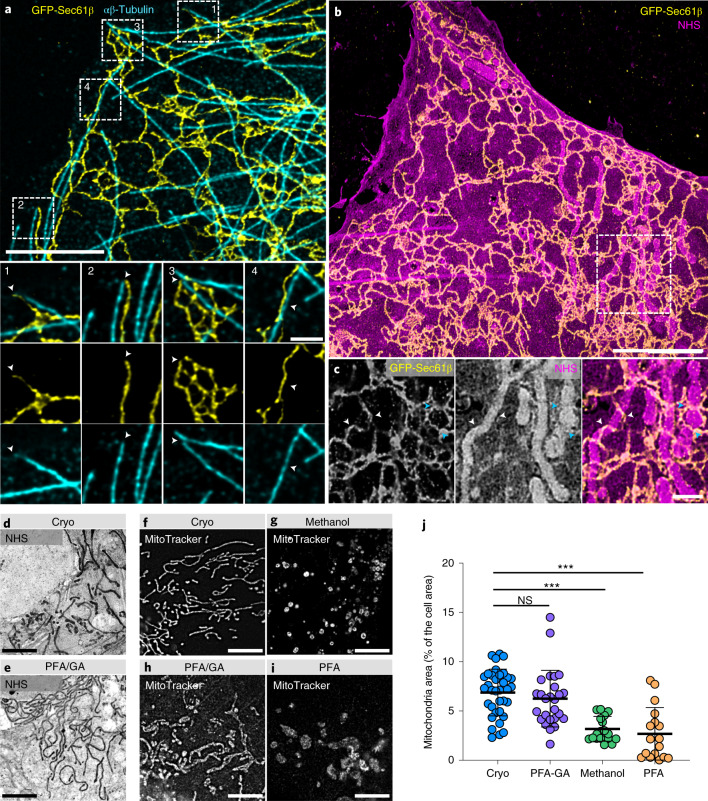
Cryo-ExM can capture fine membrane-based structures. **a**, Confocal image of cryofixed, expanded U2OS cell expressing the ER marker GFP-Sec61β stained with α/β-tubulin (cyan) and GFP (yellow). White dashed squares indicate the position of insets (1–4) showing the ER–MT contacts (white arrowheads). Scale bars, 2.5 μm, 500 nm (insets). **b**, Confocal image of cryofixed, expanded U2OS cell expressing the ER marker GFP-Sec61β stained for GFP (yellow) and NHS-ester (magenta). Scale bar, 5 μm (**c**). Inset from the region depicted by a white dashed square shown in **b**. ER-mediated wrapping and entanglement of mitochondria are indicated by white and blue arrowheads respectively. Scale bar, 1 μm. **d**,**e**, Confocal images of cryofixed (**d**) and PFA/GA-fixed (**e**) U2OS cells, expanded and stained with NHS-ester (NHS, gray). Scale bar, 5 μm. **f**–**i**, Widefield images of expanded U2OS cells incubated with MitoTracker to stain the mitochondrial matrix after cryo (**f**), methanol (**g**), PFA/GA (**h**) or PFA (**i**) fixations. Scale bar, 5 μm. **j**, Quantification of mitochondrial area (% of the cell area) in cryofixed (blue dots), PFA/GA (purple dots), methanol (green dots), PFA (orange dots) cells after expansion. Mean ± s.d., cryo, 6.9 ± 2.4%; PFA/GA, 6.3 ± 2.8%; methanol, 3.2 ± 1.3; PFA, 2.9 ± 2.7%. *n* = 35, 26, 19 and 17 for cryo, PFA/GA, methanol and PFA, respectively (cryo versus PFA/GA *P* > 0.999; cryo versus methanol *P* < 0.0001; cryo versus PFA *P* < 0.0001; one-way-analysis of variance (ANOVA) followed by Kruskal–Wallis test). NS, not significant. Source data

**Extended Data Fig. 3:**
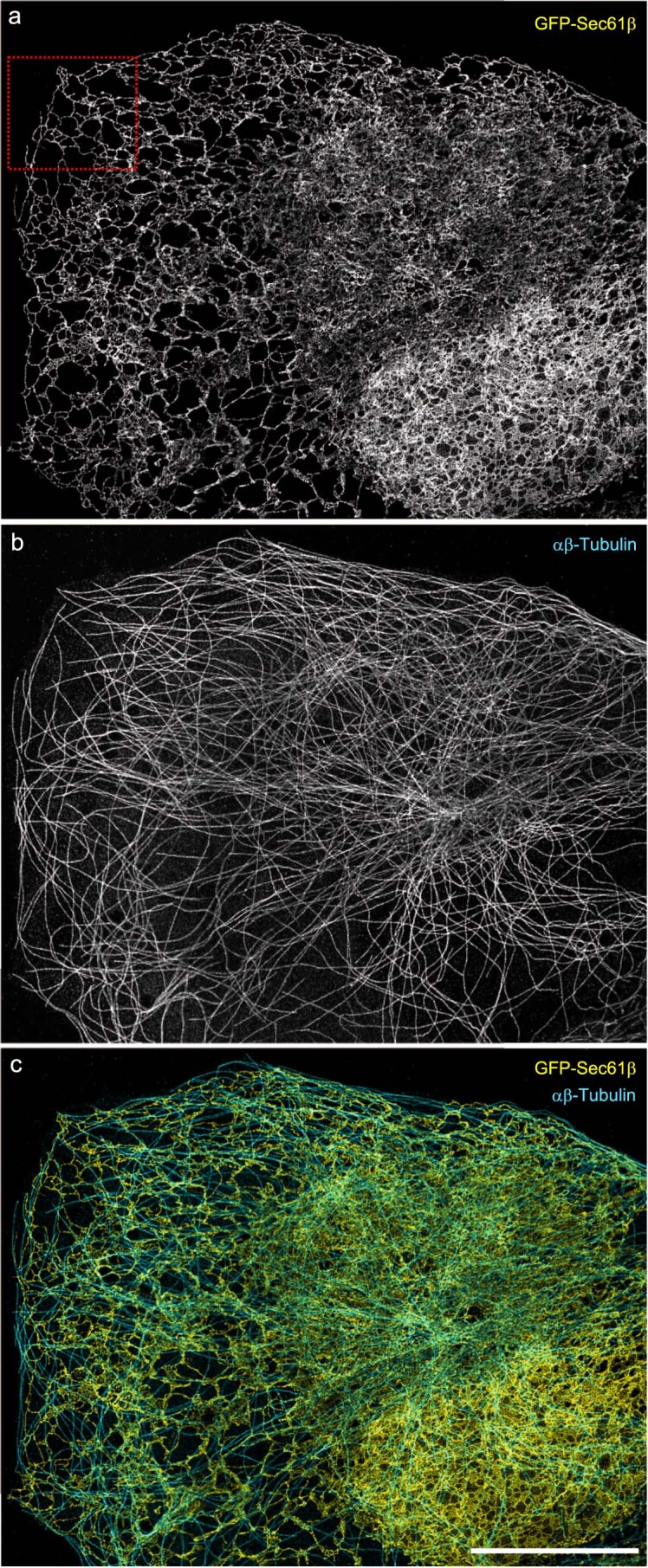
Full image of the inset shown in Fig. 2a. (**a-c**) Confocal image of cryo-fixed, expanded U2OS cell expressing the ER marker GFP-Sec61β stained with GFP (yellow) (**a, c**) and α/β-tubulin (cyan) (**b**, **c**). The region shown in Fig. 2a is depicted by the red dashed square. Scale bar = 10 μm.

**Extended Data Fig. 4:**
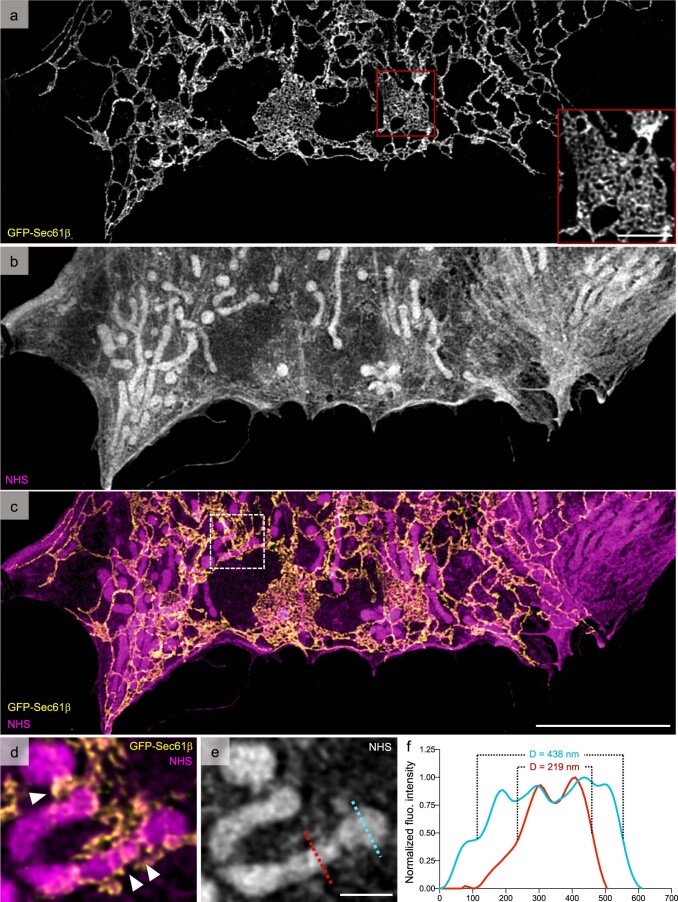
ER-mitochondria contact-sites observed by cryo-ExM. (**a-c**) Confocal image of cryo-fixed U2OS cell expressing the ER marker GFP-Sec61β, expanded and stained with GFP (yellow) (**a, c**) and NHS-ester (magenta) (**b**, **c**). Inset in panel (**a**) shows the excellent preservation of ER sheet after cryo-fixation. Scale bars = 5 μm and 1 μm (inset). (**d**,**e**) Insets of the region depicted by a white dashed square on panel **c**, showing the ER-mitochondria contact sites. Arrowheads indicate the ER-mediated constriction of a mitochondrion. Scale bar = 500 nm. (**f**) Plot profile of the indicated cross-section in (**e**) showing the reduction of the mitochondrion diameter at the position of the ER contact site.

From these results, we next investigated whether the ER–MT interactions, previously observed using live SRM imaging^15^, could be captured using Cryo-ExM (Fig. 2a and Extended Data Fig. 3). Thus, we looked at cell edges and found individual ER tubules in contact with microtubules, resembling the described ER attachment to the tips of polymerizing MT^15^ (insets 1–3) and ER branching along the MT (inset 4) (Fig. 2a). Moreover, we found that cryo-ExM can retain and resolve the nanoscale-sized holes in ER sheets as observed previously in live-STED imaging^17^ (Fig. 2a,b and Extended Data Figs. 3 and 4a, inset). Besides microtubules, ER has been also involved in the regulation of mitochondrial function and ER-mitochondria contact sites have been extensively studied notably by live SRM^15^. Hence, using NHS-ester staining^18^, a compound that reacts with the primary amines of proteins, we performed a global proteome labeling, consistently revealing the position of mitochondria in the global cellular context^18,19^. Combining NHS-ester to ER labeling, we could also observe that cryo-ExM can capture ER wrapping as well as entanglement of the mitochondria (Fig. 2b,c and Extended Data Fig. 4). Finally, the use of MitoTracker allowed us to demonstrate that both cryo and PFA/GA fixations preserve better the ultrastructure of mitochondria compared to methanol or PFA (Fig. 2d–j).

### Cryo-ExM highly preserves the cytoskeleton landscape

**Fig. 3:**
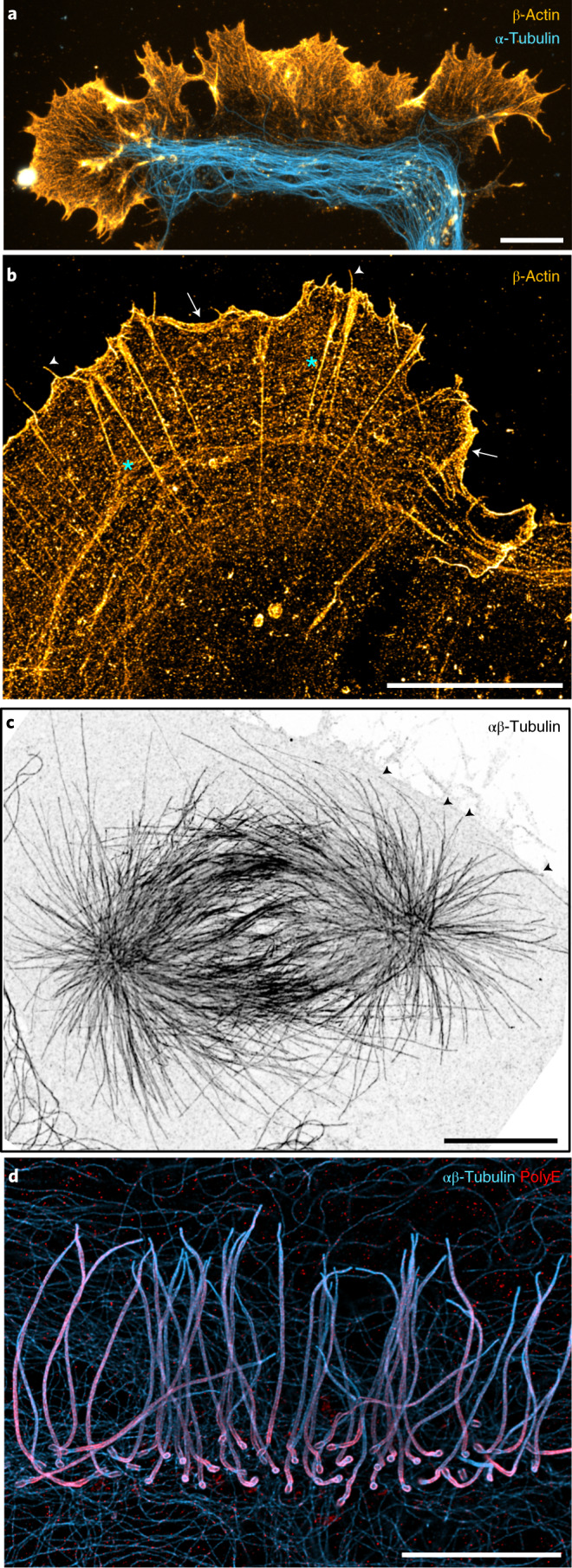
Cryo-ExM preserves cytoskeleton elements. **a**, Widefield image of cryofixed, expanded neuronal growth cone stained with α-tubulin (cyan) and β-actin (orange hot) showing characteristic actin ruffles at the cell periphery and internal microtubule bundles. Scale bar, 5 μm. **b**, Confocal image of cryofixed, expanded U2OS cell stained for β-actin showing three canonical actin structures: lamellipodia (white arrows), filopodia (white arrowheads) and stress fibers (turquoise asterisks). Scale bar, 10 μm. **c**, Confocal image of cryofixed, expanded RPE-1 cell stained for α/β-tubulin (gray) showing the mitotic spindle and astral microtubules preservation. Black arrowheads depict the contact of the astral microtubule with the cell cortex. Scale bar, 5 μm. **d**, Confocal image of cryofixed, expanded multiciliated ependymal cell stained for α/β-tubulin (cyan) and poly-glutamination (PolyE) (red). Scale bar, 5 μm.

**Extended Data Fig. 5:**
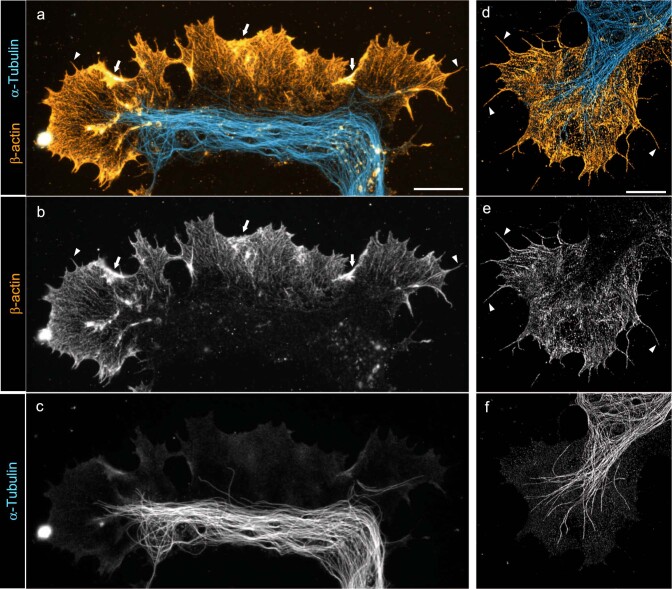
Neuronal growth cones observed by cryo-ExM. Widefield **(a-c)** and confocal images (**d-f**) of cryo-fixed, expanded neuronal growth cone from hippocampal cultured neurons stained with α-tubulin (cyan) and β-actin (orange hot) showing characteristic actin ruffles (arrows) and filopodia (arrowheads) at the cell periphery and internal microtubule bundles. Scale bar = 2.5 μm.

We further explored the preservation of the cytoskeletal landscape. First, we looked at the actin network of growth cones in cultured hippocampal neurons, known for their unique cytoskeleton organization^20^. By simultaneously imaging the actin cytoskeleton using β-actin antibodies together with microtubules, we found that both cytoskeletons remain intact in cryo-ExM and their canonical organization is preserved, with internal microtubule bundles and actin structures such as filopodia and ruffles forming waves at the cell periphery (Fig. 3a and Extended Data Fig. 5). Second, we stained U2OS cells for actin and could unveil the different typical actin networks, the lamellipodia, filopodia and stress fibers^21^ (Fig. 3b). Finally, we turned to LifeAct to label filamentous actin^22^ and analyzed it under different fixation conditions (Supplementary Fig. 1). Notably, we found that cryo-ExM gave similar results as the gold standard PFA/GA for actin and did not affect expansion as we noticed minimal distortions of 1.6%, similar or smaller to the distortions observed using other expansion microscopy methods^1,10^ (Supplementary Fig. 2).

**Extended Data Fig. 6:**
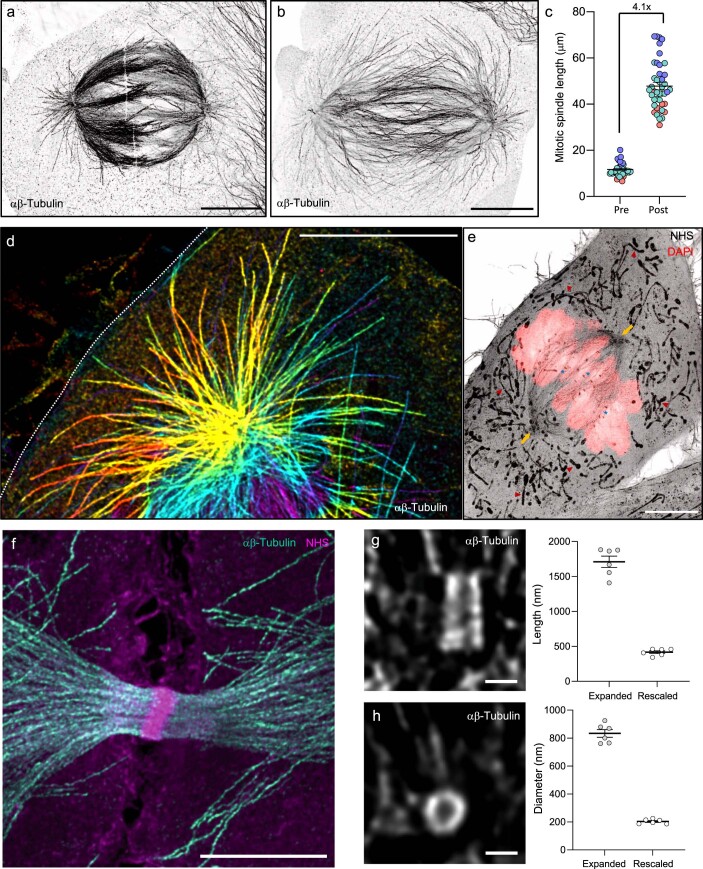
Mitotic events observed by cryo-ExM. (**a, b**) Confocal images of cryo-fixed and expanded MEF (**a**) and RPE-1 (**b**) cells in mitosis, stained with α/β-tubulin. Scale bar= 5 μm. (**c**) Measurement of the mitotic spindle length from non expanded cells (Pre) and expanded cells (Post). The expansion factor was calculated as follow: the average spindle length of expanded cells divided by the average spindle length of non expanded cells. Average +/− SD: Pre= 11.71 ± 2.8 μm; Post= 47.84 ± 10.44 μm. N = 37 and 43 for Pre and Post expanded cells respectively (Orange: prophase; green: metaphase; purple: anaphase). (**d**) Inset from Fig. 3c shows the preservation of the astral microtubule touching the cell cortex (white dashed line). Color code indicates the z-position. Scale bar = 5 μm. (**e**) Confocal image of a mitotic MEF cell stained for NHS-ester (grey) and DAPI (red) allowing the visualization of the kinetochore attached to chromosomes (blue stars), the mitotic spindle pole (yellow arrows), and the mitochondria (red arrowheads). Scale bar= 5 μm. (**f**) Z-projection of a mitotic MEF cell stained for α/β-tubulin (green) and NHS-ester (magenta) showing the preservation of the midbody. Scale bar = 2.5 μm. (**g, h**) Single plan images of mitotic centrioles stained for α/β-tubulin. Scale bar= 200 nm. Quantifications of the length (**g**) and diameter (**h**) of mitotic centrioles witnessing the 4-fold isotropic expansion of the mitotic cells. ‘Expanded’ indicates the length and diameter of centrioles measured after expansion. ‘Rescaled’ indicates the length and diameter of centrioles after applying the expansion factor (see methods) Average +/− SD: Length: 1709 ± 199 nm, 417 ± 48 nm; Diameter: 833 ± 67 nm, 203 ± 16 nm. N = 6 centrioles from 3 independent experiments. Source data

**Extended Data Fig. 7:**
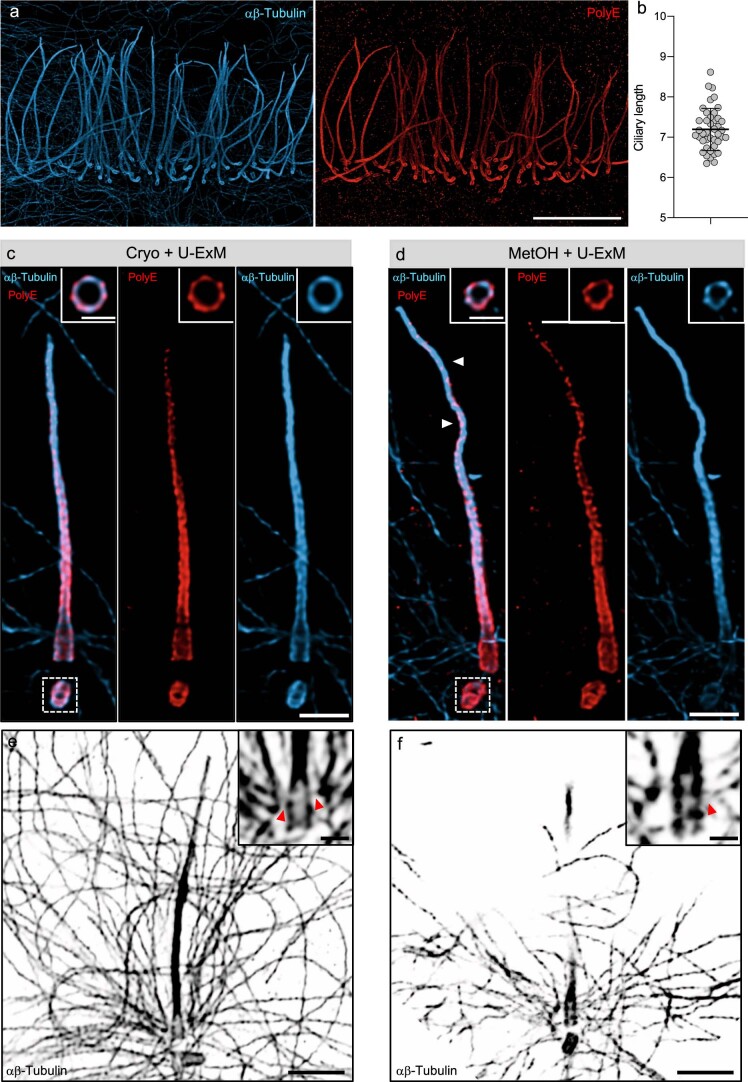
Motile and primary cilia observed by Cryo-ExM. (**a**) Confocal image of cryo-fixed multiciliated ependymal cell, expanded and stained for α/β-tubulin (cyan) and poly-glutamination (PolyE) (red). Scale bar= 5 μm. (**b**) Quantification of the length of motile cilia from differentiated multiciliated ependymal cells. Average +/− SD: 7.19 ± 0.52 μm (N = 42 cilia from 3 gels). (**c, d**) Confocal images of cryo-fixed (**c**) or Methanol-fixed (**d**), expanded RPE-1 cell stained with α/β-tubulin (cyan) and PolyE (red) showing better preservation of the primary cilium architecture (white arrowheads indicate a wavy cilium) with cryo-fixation. Inset shows a single plan of the daughter centriole in top view. Scale bars= 500 nm and 200 nm (inset). (**e, f**) Confocal images of cryo-fixed (**e**) or Methanol-fixed (**f**), expanded RPE-1 cell stained with α/β-tubulin showing the better preservation of the surrounding microtubules and their attachment to basal body (inset, red arrowheads) in cryo-fixed cells. Scale bars = 1 μm and 200 nm (inset). Source data

We also analyzed the microtubule cytoskeleton organization. We first inspected the highly dynamic microtubule network found in mitotic RPE-1 cells and observed that cryo-ExM enabled high preservation of the mitotic spindle as well as astral microtubules, which are difficult to maintain owing to the chemical fixations artifacts^23^ (Fig. 3c and Extended Data Fig. 6a–e). We also found that the mitotic spindle displayed an isotropic ~fourfold expansion by measuring the spindle length before and after expansion as well as the centriole as an internal ruler^11^ (Extended Data Fig. 6a–c,g,h). Using NHS-ester staining^18^, we observed that cryo-ExM protects the overall organization of the mitotic cells as chromosomes, intact mitochondria and the midbody could be observed (Extended Data Fig. 6e,f). Second, we looked at nondividing cells where cilia protrude from the cell surface and found that motile cilia from ependymal cells were fully preserved, displaying the canonical length of 7 μm^24^ (Fig. 3d and Extended Data Fig. 7a,b), as well as primary cilia where we detected that microtubule nucleation sites on the underlying mature basal body^25^ were overall better preserved than with regular methanol fixation (Extended Data Fig. 7c–f).

### Cryo-ExM improves epitope accessibility

**Fig. 4:**
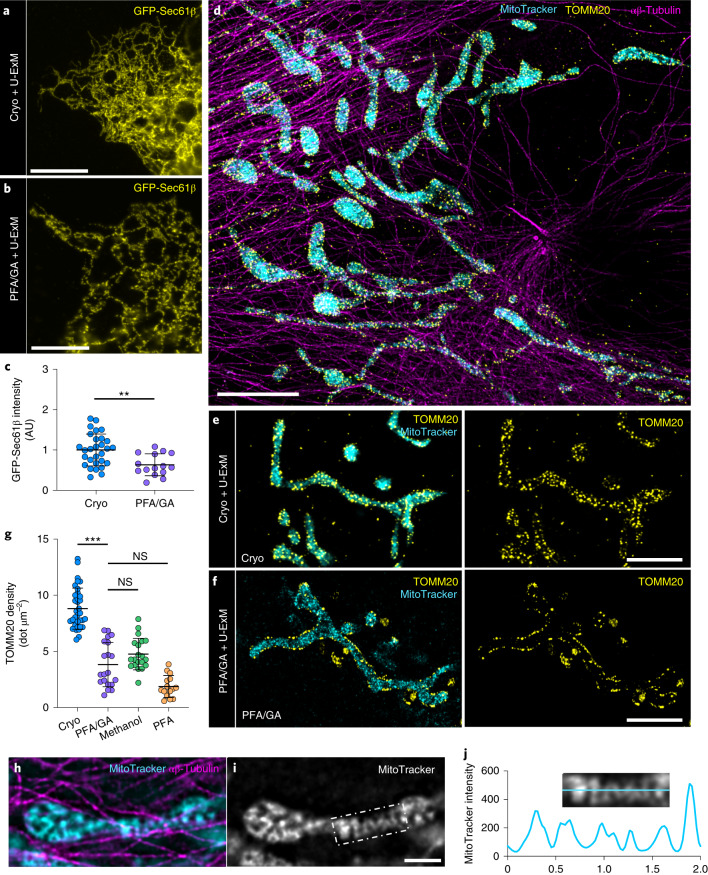
Cryo-ExM preserves epitopes. **a**,**b**, Widefield images of expanded U2OS cells expressing GFP-Sec61β stained for GFP (yellow) after cryofixation (**a**) or PFA/GA fixation (**b**). Scale bar, 5 μm. **c**, Quantification of GFP-Sec61β intensity in expanded U2OS after cryofixation or PFA/GA fixation. Mean ± s.d., cryo, 1 ± 0.39; PFA/GA, 0.63 ± 0.27 (*n* = 29 and 15 cells for cryo and PFA/GA, respectively, from three independent experiments, *P* = 0.0025, two-sided Student’s *t*-test). AU, arbitrary units. **d**, Confocal image of cryo-fixed expanded RPE-1 cell stained with MitoTracker (cyan) and TOMM20 (yellow) to visualize the mitochondrial matrix and outer membrane, respectively and α/β-tubulin (magenta). Scale bar, 5 μm. **e**,**f**, Single/double channel images showing mitochondrial TOMM20 staining on expanded RPE-1 cells after cryofixation (**e**) or PFA/GA fixation (**f**). Scale bar, 2 μm. **g**, Quantification of TOMM20 density (number of TOMM20-positive dots per μm^2^) in cryofixed (blue dots), PFA/GA (purple dots), methanol (green dots), PFA (orange dots) cells after expansion. Mean ± s.d., cryo, 8.8 ± 1.8 dots μm^−^^2^; PFA/GA, 3.8 ± 1.9 dots μm^−2^; methanol, 4.8 ± 1.4 dots μm^−2^; PFA, 1.9 ± 0.9 dots μm^−2^ (*n* = 35, 20, 20 and 15 for cryo, PFA/GA, methanol and PFA, respectively. Cryo versus PFA/GA *P* < 0.0001; PFA/GA versus methanol *P* > 0.999; PFA/GA versus PFA *P* = 0.303, one-way-ANOVA followed by Kruskal–Wallis test). **h**–**j**, Widefield image of cryofixed, expanded RPE-1 cell stained with α/β-tubulin (magenta) and MitoTracker (cyan). The dashed square (**i**) indicates the area used for the plot profile (**j**) across mitochondria highlighting the position of the mitochondrial cristae. Scale bar, 1 μm. Source data

**Extended Data Fig. 8:**
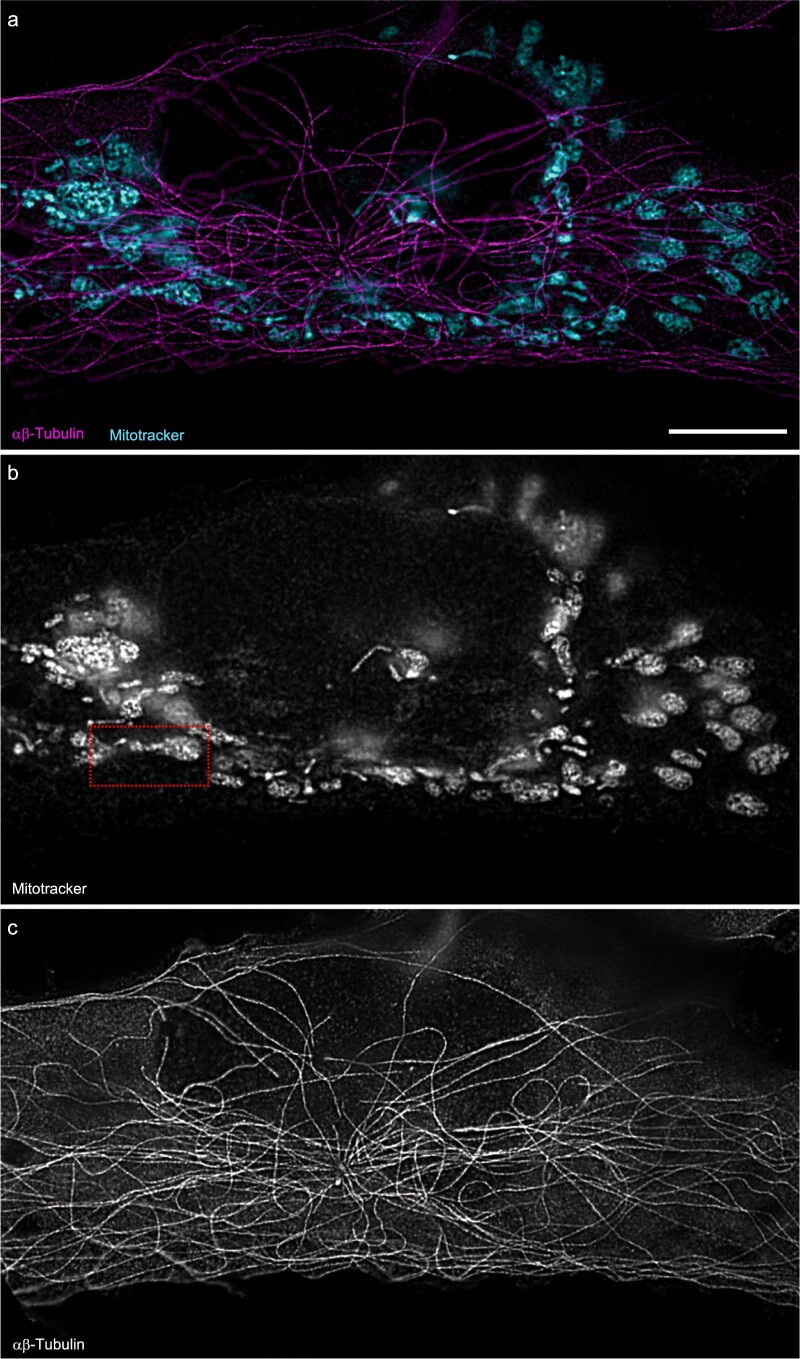
Full image of the inset shown in Fig. 4h, i. (**a**-**c**) Widefield image of cryo-fixed RPE-1 cell, expanded and stained with α/β-tubulin (**a**, **c**) and mitotracker (**b**, **c**). The region shown in Fig. 4 (**h**, **i**) is depicted by the red dashed square. Scale bar= 5 μm.

Next, as it is known that chemical fixations can affect epitopes accessibility in immunostainings^3,5^, we investigated whether Cryo-ExM could alleviate this issue. To do so, we first compared fixation effects on the staining intensity of the ER using GFP-Sec61β as a proxy. We found that PFA/GA fixation decreased overall fluorescence intensity by 40% compared to Cryo-ExM (Fig. 4a–c). Then, we analyzed the fixation effect on the outer mitochondrial membrane translocase TOMM20 density (Fig. 4d–g). Notably we observed a greater labeling density using cryofixation compared to PFA/GA, PFA alone or methanol (Fig. 4g). We also noticed that the use of MitoTracker allowed us to resolve the mitochondrial cristae, highlighting that the inner architecture of this organelle is intact (Fig. 4h–j and Extended Data Fig. 8). Also, we noticed that when staining microtubules and mitochondria together, the cytoplasmic signal for tubulin was absent in the space occupied by mitochondria (Supplementary Fig. 3). We hypothesize that this could correspond to the cytoplasmic soluble pool of tubulin that is usually precipitated or lost owing to chemical fixation and permeabilization^3^.

### Versatility of the Cryo-ExM method

**Fig. 5:**
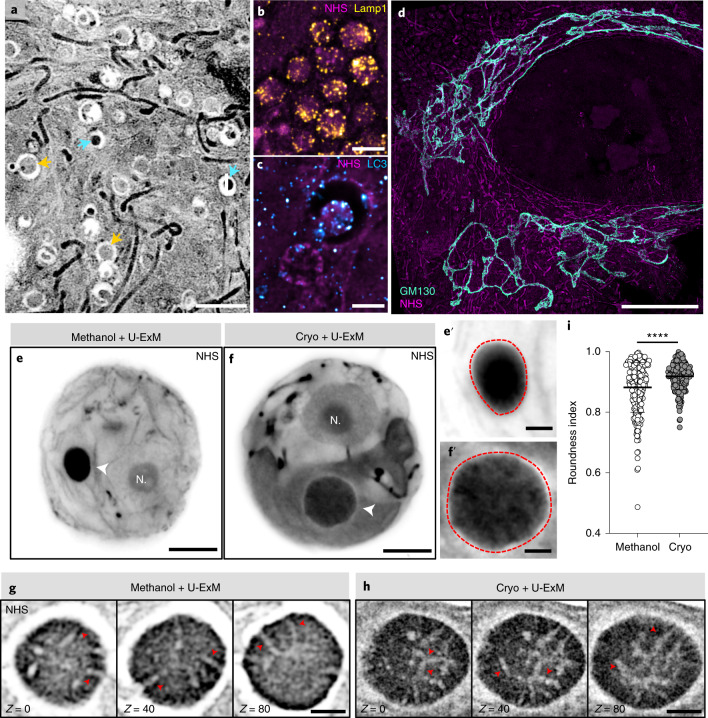
Versatility of Cryo-ExM. **a**–**c**, Confocal image of a cryofixed RPE-1 cell, expanded and stained for NHS-ester (gray). Blue and yellow arrows indicate structures that look like lysosomes and autophagosomes, respectively. Scale bar, 2 μm. Widefield images of cryofixed starved RPE-1 cells, expanded and stained for NHS-ester with either the lysosomal marker Lamp1 (**b**, yellow) or the autophagosome marker LC3 (**c**, cyan). Scale bar, 1 μm. **d**, Confocal image of a cryofixed U2OS cell, expanded and stained for NHS-ester (magenta) and the Golgi marker GM130 (fresh green). Scale bar, 10 μm. **e**-**fʹ**, Widefield images of methanol-fixed (**e**,**eʹ**) or cryofixed (**f**,**fʹ**) *C.* *reinhardtii* cell expanded and stained with NHS-ester, which reveals the entire cellular context (N, nucleus). White arrowheads indicate the pyrenoid (**e**,**f**). Scale bars, 2 μm (**e**,**f**) and 500 nm (**eʹ**,**fʹ**). **g**,**h**, Z-sections across a methanol-fixed (**g**) and cryofixed (**h**) *C.* *reinhardtii* cell expanded and stained with NHS-ester unveiling a density that we hypothesized to be pyrenoid tubules inside the phase-separated organelle (red arrowheads). Scale bar, 500 nm. **i**, Quantification of the pyrenoid roundness showing a better preservation with cryofixation compared to methanol fixation. The roundness was manually traced as shown by the dashed red lines in **eʹ**,**fʹ**. Mean ± s.d., cryo, 0.92 ± 0.04; methanol, 0.88 ± 0.08 (*n* = 247 and 240 cells for cryo and methanol, respectively, from four independent experiments; *P* < 0.0001, two-sided Mann–Whitney *U*-test). Source data

**Extended Data Fig. 9:**
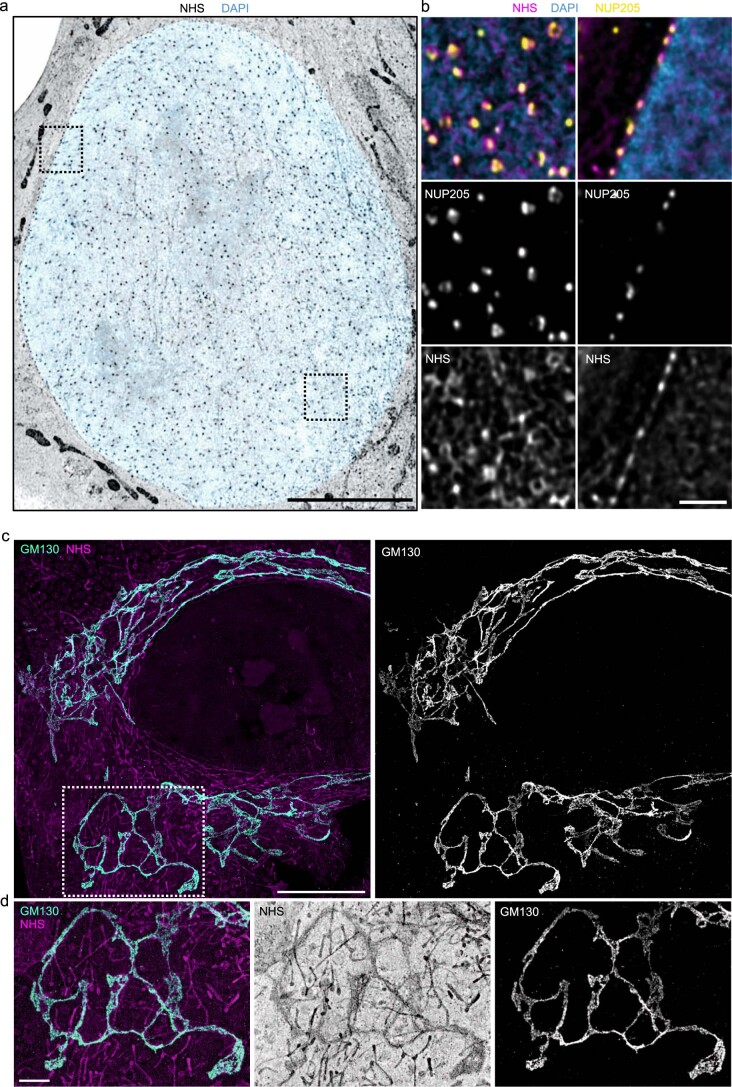
Visualization of nuclear pores and golgi apparatus by cryo-ExM. (**a**) Confocal image of cryo-fixed U2OS cells, expanded and stained for NHS-ester (grey) and DAPI (cyan). Scale bar= 5 μm. (**b**) Insets from the region depicted in (**a**) by the black dashed squares. Top view (left) and side view (right) of nuclear pores stained with NHS-ester (magenta), NUP205 (yellow) and DAPI (blue). Scale bar= 500 nm. (**c**) Confocal image of a cryo-fixed U2OS cell, expanded and stained for NHS-ester (magenta) and the golgi marker GM130 (fresh green). Scale bar= 10 μm. (**d**) Insets from the region depicted in (**c**) by the white dashed squares showing the organization of the Golgi apparatus. Scale bar= 2 μm.

Finally, we investigated the generality of epitope preservation of Cryo-ExM by assessing other cellular structures such as lysosomes/autophagosomes (Lamp1 and LC3), Golgi apparatus (GM130) and nuclear pores (NUP205). We found that all structures could be visualized in Cryo-ExM, demonstrating the wide range of epitope preservation of this method (Fig. 5a–d and Extended Data Fig. 9). In addition, we assessed whether cryofixation can solve two well-known artifacts of aldehydes fixations: the exclusion of the transcription factor SOX2 from the DNA in mitosis^26^ and the cellular distribution of the cell surface glycoprotein CD44^27^. Probably owing to the instantaneous fixation that prevents protein diffusion, we found that cryofixation preserves the correct localization of SOX2 on chromatin of nonexpanded human embryonic kidney (HEK) cells (Supplementary Fig. 4a,b) and that CD44 labeling is highly preserved with a pattern colocalizing with actin fibers as previously observed in two-color SRM^28^ as well as in consistency with the known colocalization of CD44 with the Golgi apparatus (Supplementary Fig. 4c–f).

**Extended Data Fig. 10:**
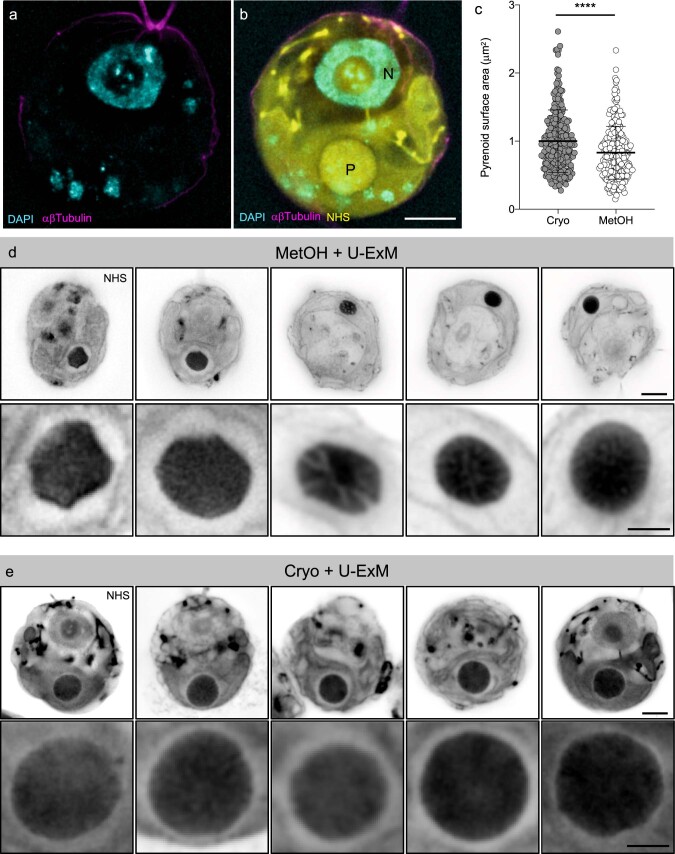
Phase-separated organelle in cryo-ExM. (**a**, **b**) Widefield image of the cryo-fixed and expanded *C. reinhardtii* cell shown in Fig. 5f, stained for DAPI (cyan) (**a**, **b**), α/β-tubulin (magenta) (**a**, **b**) and NHS-ester (yellow) (**b**) that reveals the cellular context. N = nucleus, P = pyrenoid. Scale bar = 2 μm. (**c**) Quantification of the pyrenoid area from cryo-fixed and Methanol-fixed (MetOH) expanded *C. reinhardtii* cells. Average +/− SD: Cryo= 1.00 ± 0.45, MetOH= 0.8 ± 0.38 (N = 270 and 264 for Cryo and MetOH, respectively, from 4 independent experiments; p < 0.0001, two-sided Mann Whitney test). (**d**, **e**) Gallery of MetOH-fixed (**d**) and cryo-fixed (**e**) *C. reinhardtii* cells, expanded and stained for NHS-ester. Insets show the pyrenoid of each cell. Scale bars= 2 μm and 500 nm (inset). Note that the *Chlamydomonas* cells fixed with methanol seems to display a different morphology based on the NHS-ester staining compared to cryo-fixation that could be due to the fixation itself. Source data

Last, we further investigated the ability of Cryo-ExM to safeguard the native cellular organization by imaging a soft organelle that is affected by chemical fixation, namely phase-separated organelles^29^. Therefore, we turned to analyze the pyrenoid, a liquid-like droplet organelle from the green algae *Chlamydomonas* *reinhardtii*, made of the densely packed CO_2_-fixing enzyme Rubisco, crucial for the photosynthesis process^29^. Using NHS-ester staining^18,19^, we observed that both methanol and cryofixation could preserve structures inside the pyrenoid that most likely correspond to the pyrenoid tubules, previously observed by cryo-electron tomography after cryo-FIB-milling^29^ (Fig. 5e–h). However, we noticed that upon methanol fixation, the expanded *Chlamydomonas* cells were slightly collapsed and their pyrenoid shape was variable, as indicated by the roundness index and the area (Fig. 5e,eʹ,i and Extended Data Fig. 10). As phase-separated organelles are often perfect spherical droplet^30^, this result might indicate that methanol fixation induces a protein precipitation deforming the pyrenoid. In contrast, we found that applying Cryo-ExM directly on *Chlamydomonas* cells seems to better preserve the liquid-droplet shape of the pyrenoid as these showed more homogeneous circularity (Fig. 5f,fʹ,i and Extended Data Fig. 10), suggesting that these cellular structures formed by phase separation remain intact under these conditions.

## Discussion

In this work, we introduce a new method to perform SRM by coupling cryofixation of a biological specimen with ExM. With this, we provide a universal framework to visualize subcellular compartments without chemical fixation artifacts such as structural alteration and loss of antigenicity. Moreover, Cryo-ExM alleviates the issues of optimizing fixation conditions required to visualize specific structures and is therefore expected to enable more accurate protein localization. Notably, this method also demonstrates that the classical cryo-substitution protocols developed for EM are compatible with expansion microscopy by replacing the EM resin with hydrogel monomer solutions. Therefore, this approach may also be applicable on tissues cryofixed by high-pressure freezing as well as in hydrogel-based tissue clearing. Finally, as expansion microscopy is also compatible with SIM, STED or *d*STORM^31–33^, our method now allows all these microscopy modalities to image easily cells in their native state, paving the way for further studies of complex cellular processes.

## Methods

### Reagents and reagent preparation

The following reagents were used in this study: Glutaraldehyde (GA, 25%, G5882, Sigma), Paraformaldehyde (PFA, 16%, cat. no. 15710, EMS), methanol (99.9%, M/4058/17, Thermo Fisher Scientific), ethane:propane 37%:63% (PanGas), acetone (99.8% AcroSeal, cat. no. 67-64-1, Acros Organics), Formaldehyde (FA, 36.5–38%, F8775, SIGMA), Acrylamide (AA, 40%, A4058, SIGMA), N,Nʹ-methylenbisacrylamide (2%, M1533, SIGMA), Sodium acrylate (SA, 97–99%, 408220, SIGMA), ammonium persulfate (APS, 17874, Thermo Fisher Scientific), tetramethylethylendiamine (TEMED, 17919, Thermo Fisher Scientific), nuclease-free water (AM9937, Ambion-Thermo Fisher Scientific), and poly-d-lysine (A3890401, Gibco).

SA stock solution was prepared by resuspending 25 g of SA powder in 40.8 g of nuclease-free water (final concentration 38% w/w). SA was added in three times under agitation and left steering at 4 °C overnight until complete dissolution. Monomer solution is composed of 19% SA, 10% AA and 0.1% N,Nʹ-methylenbisacrylamide completed with 10× PBS and stored at −20 °C at least 24 h before gelation. Denaturation buffer is composed of 200 mM SDS, 200 mM NaCl and 50 mM Tris-BASE, pH 9.

### Cell culture

#### *Chlamydomonasreinhardtii*

The cell-wall-less *Chlamydomonas* strain CW15 was grown in liquid Tris acetate phosphate medium (containing Trace) at 22 °C or on Tris acetate phosphate plates with 1.5% agar, as previously described^34^. Cells were either fixed by immersion in −20 °C chilled methanol for 5 min or cryofixed with plunging in liquid ethane/propane mix (see below).

#### Cell lines

Mouse embryonic fibroblast (MEF), retinal pigment epithelium (RPE-1), *Homo* *sapiens* bone osteosarcoma (U2OS) and human embryonic kidney (HEK) cells were grown in Dulbecco’s modified Eagle’s medium and GlutaMAX (Life Technologies), supplemented with 10% fetal calf serum (Life Technologies) and penicillin and streptomycin (100 μg ml^−1^) at 37 °C in a humidified 5% CO_2_ incubator. Cells were plated at 35,000 cells cm^−2^ for U2OS and RPE-1 and at 10,000 cells cm^−2^ for MEF cells. For analysis of cilia morphology (Extended Data Fig. 7) and lysosomes/autophagosomes (Fig. 5), RPE-1 cells were incubated for 48 h in starvation medium (DMEM GlutaMAX supplemented with 0.5% fetal calf serum). For analysis of mitochondrial morphology (Figs. 2d–j and 4d–j, Extended Data Fig. 8 and Supplementary Fig. 3), cells were incubated with 100 nM MitoTracker deep red (M22426, Thermo Fisher Scientific) for 40 min before fixation.

#### Mouse neuronal cell culture

Primary cultures of hippocampal neurons were obtained according to the procedure described previously^35^. Hippocampi were dissected from E18.5 mouse embryos in HBSS (Invitrogen) containing HEPES 10 mM, streptomycin 10 µg ml^−1^, penicillin 10 U ml^−1^, treated with 0.25% trypsin-EDTA for 10 min at 37 °C and disrupted by 10–15 aspirations/ejections through a 5-ml pipette, followed by ten cycles through a micropipette tip. Dissociated hippocampal neurons were seeded in DMEM (Invitrogen) supplemented with 10% heat-inactivated horse serum at 50,000 cells cm^−2^ in six-well plates on 12-mm glass coverslips precoated overnight with 50 μg ml^−1^ poly-d-lysine (Sigma) at 37 °C. At 20 h after seeding, the medium was changed to the culture medium (Neurobasal (Invitrogen), B27 supplement 2%, sodium pyruvate 1 mM, l-glutamine 2 mM, streptomycin 10 µg ml^−1^, penicillin 10 U ml^−1^) and neurons were imaged between 2–4 d in vitro. These experiments were carried out in accordance with the Institutional Animal Care and Use Committee of the University of Geneva and with permission of the Geneva cantonal authorities.

#### Mouse ependymal cell culture

Ependymal cell culture was prepared as previously described^36^. Newborn mice (P0–P3) were killed by decapitation, and brains dissected in Hank’s solution (10% HBSS, 5% HEPES, 5% sodium bicarbonate and 1% penicillin/streptomycin (P/S)). The extracted ventricular walls were cut manually into pieces, followed by enzymatic digestion (DMEM GlutaMAX, 33% papain (Worthington 3126), 17% DNase at 10 mg ml^−1^ and 42% cysteine at 12 mg ml^−1^) for 45 min at 37 °C in a humidified 5% CO_2_ incubator. Digestion was stopped by the addition of a solution of trypsin inhibitors (Leibovitz Medium L15, 10% ovomucoid at 1 mg ml^−1^ and 2% DNase at 10 mg ml^−1^). Cells were washed and resuspended in DMEM GlutaMAX supplemented with 10% fetal bovine serum and 1% P/S in a poly-d-lysine-coated flask. Ependymal progenitors proliferated for 5 d until confluence followed by shaking (250 r.p.m.) overnight. Pure confluent astroglial monolayers were replated at a density of 7 × 10^4^ cells per cm^2^ in DMEM GlutaMAX, 10% fetal bovine serum, 1% P/S on poly-d-lysine-coated coverslips. After 24 h, the medium was replaced by serum-free DMEM GlutaMAX 1% P/S, to trigger gradual ependymal differentiation (2–3 d in vitro).

### Cloning and GFP-construct overexpression

#### GFP-Sec61β

The truncated form of Sec61β(45-97) was generated according to previous work^37^, as the authors found that this Sec61β fragment decorated the ER without affecting microtubule bundling. GFP-Sec61β (133–291) was obtained by subcloning the C-terminal DNA fragment (133–291) (corresponding to aa 45 to 97) from the pAc-GFPC1-Sec61β (plasmid 15108 Addgene) into the peGFP-C1 vector, using the following primers: Fwd (EcoRI) 5ʹ- ATgaattctGGCCGCACAACCTCG-3ʹ and Rev (ApaI) 5ʹ- ATTTgggcccCTACGAACGAGTGTACTTGCC-3ʹ. This cloning was performed by the Aumeier Laboratory (University of Geneva).

#### LifeAct-GFP

LifeAct-eGFP plasmid (Addgene no. 58470) was a kind gift from R. Sadoul.

U2OS expressing GFP-sec61β (133–291) or LifeAct-GFP were transiently transfected with JetPRIME following the manufacturer’s instructions. After 24 h of expression, cells were fixed as described below.

### Cell fixation

Cells grown at desired confluence were washed in PBS and either cryofixed (see below) or with the following protocols when specified: (1) by immersion in −20 °C chilled methanol for 5 min, (2) in 4% PFA for 15 min at room temperature, (3) in 3% PFA + 0.1% GA for 20 min at room temperature or (4) in 1% PFA + 0.2% GA (Supplementary Fig. 4c–f) for 20 min at room temperature.

### Cryo-ExM protocol

#### Plunge-freezing and freeze substitution

The 12-mm coverslips containing the sample were held halfway with a thin tweezer (Dumont 5, Sigma F6521-1EA), the excess of remaining medium was strongly blotted with a filter paper and coverslips were rapidly plunged with a homemade plunge freezer into liquid ethane or an ethane/propane mix cooled with liquid nitrogen (Extended Data Fig. 1a–c). Note that the homemade plunger is a classical system used by most of the cryomicroscopy laboratories but an automatic system might work similarly. No difference could be observed between pure ethane and an ethane/propane mix, the latter mix being more convenient because it does not solidify at the temperature of liquid nitrogen^38^. Coverslips were then rapidly transferred into a 5-ml Eppendorf tube containing 2.5 ml of liquid nitrogen-chilled acetone (Extended Data Fig. 1d). Tubes were placed on dry ice with a 45° angle and agitated overnight to allow the temperature to rise to −80 °C (Extended Data Fig. 1d). Samples were further incubated without dry ice for 1.5 h until the temperature reached ~0 °C. Samples were then rehydrated in successive ethanol:water baths, 5 min each, as follows: ethanol 100%, ethanol 100%, ethanol 95%, ethanol 95%, ethanol 70%, ethanol 50% and PBS. Cells were stored in PBS until expansion or directly processed for immunostaining (Extended Data Fig. 6c and Supplementary Figs. 2 and 4a,b).

#### Ultrastructure expansion microscopy

Expansion of the cells was performed as previously described^39^. Briefly, fixed cells (cryo, PFA, PFA/GA or methanol) were incubated for 3 to 5 h in 2% AA and 1.4% FA diluted in PBS at 37 °C before gelation in monomer solution containing 0.5% tetramethylethylendiamine and ammonium persulfate. Next, cells were incubated for 5 min on ice followed by 1 h at 37 °C and incubated for 1.5 h at 95 °C in denaturation buffer. Gels were washed twice in ddH_2_O. Note that the original U-ExM protocol without previous fixation^10^ depolymerizes cytoplasmic microtubules^11^. In contrast, cryofixation before U-ExM protocol preserves cytoplasmic microtubules.

Note that the quality of sample preservation using the plunger was also compared to manual immersion. As shown in Supplementary Fig. 5a–d, manual immersion leads to wavy and broken microtubules, whereas the plunger fully preserves their native structures. Note also that sample fractures could be sometimes observed, as classically observed in cryomicroscopy^40^ (Supplementary Fig. 5e–h).

### Immunostaining

Gels were incubated in PBS for 30 min and stained for 3 h at 37 °C under constant agitation with the following antibodies diluted in 2% PBS–BSA: tubulin monobodies AA344 (1:250 dilution, scFv-S11B, β-tubulin) and AA345 (1:250 dilution, scFv-F2C, α-tubulin)^41^, mouse monoclonal anti-β-actin (1:250 dilution, 60008-1-1, Proteintech), rabbit polyclonal anti-α-tubulin (1:250 dilution, ab18251, Abcam), rabbit polyclonal anti-PolyE (1:500 dilution, AG-25B-0030, AdipoGen), rabbit polyclonal anti-GFP (1:250 dilution, TP401, Torrey Pines), rabbit polyclonal anti-TOMM20 (1:250 dilution, ab186734, Abcam), rabbit polyclonal anti-GM130 (1:250 dilution, 11308-1-AP, Proteintech), rabbit polyclonal anti-actin (1:250 dilution, ab1801, Abcam), rabbit polyclonal anti-NUP205 (1:250 dilution, 24439-1-AP, Proteintech), rabbit polyclonal anti-LC3 (1:250 dilution, 14600-1-AP, Proteintech), rabbit polyclonal anti-Lamp1 (1:250 dilution, D2D11, Cell Signaling), rabbit polyclonal anti-Sox2 (1:250 dilution, 20118-1-AP,Proteintech), mouse monoclonal anti-CD44 (1:250 dilution, 60224-1-Ig, Proteintech). The following secondary antibodies were used: goat anti-rabbit Alexa Fluor 488 IgG H+L (1:400 dilution, A11008) and goat anti-mouse Alexa Fluor 568 IgG H+L (1:250 dilution, A11004) (Invitrogen, Thermo Fisher Scientific). Gels were washed three times in PBS–Tween 0.1% and expanded by successive baths of ddH_2_O. When indicated, the gel was further incubated in NHS-ester Alexa594 (20 mg ml^−1^ in PBS, AD594-31, ATTO-TEC) or NHS-ester AlexaA488 (20 mg ml^−1^ in PBS, 46402, Thermo Fisher Scientific) for 1.5 h at room temperature under constant agitation, washed three times in PBS and expanded by successive baths of ddH_2_O.

For direct immunostaining on coverslips (Extended Data Fig. 6c and Supplementary Figs. 2 and 4a,b), coverslips were incubated for 15 min in 2% PBS–BSA–Tween 0.1% and incubated with the primary antibodies diluted in 2% PBS–BSA–Tween 0.1% for 1 h at room temperature. After three washes in PBS–Tween 0.1%, secondary antibodies diluted in 2% PBS–BSA–Tween 0.1% were incubated for 1 h at temperature. Coverslips were then mounted in presence of 4,6-diamidino-2-phenylindole (DAPI) and imaged as described below. Primary and secondary antibodies were diluted twice compared to post-expansion staining.

To visualize the actin network, we either used β-actin antibodies or LifeAct-GFP. Comparison of LifeAct-GFP to β-actin staining patterns confirmed that both could be used to faithfully visualize actin in human cells using Cryo-ExM (Supplementary Fig. 1).

### Image acquisition

Pieces of gels were mounted on 24-mm round precision coverslips (1.5H, 0117640, Marienfeld) coated with poly-d-lysine for imaging. Image acquisition was performed on an inverted Leica TCS SP8 microscope or a Leica Thunder DMi8 microscope using a ×631.4 NA oil objective with Lightening or Thunder LVCC (large volume computational clearing) mode at max resolution, adaptive as ‘Strategy’ and water as ‘Mounting medium’ to generate deconvolved images. Three-dimensional stacks were acquired with 0.12 μm *z*-intervals and an *x*, *y* pixel size of 35 nm (Leica TCS SP8) or 0.21 μm *z*-intervals and an *x*, *y* pixel size of 100 nm (Thunder DMi8).

### Quantifications

For each gel, a caliper was used to accurately measure its expanded size. The gel expansion factor was obtained by dividing the expanded size by the original size of the coverslip (12 mm in this work). Each measurement was divided by the calculated expansion factor and reported as such in the graphs or figure scale bars, except in Extended Data Fig. 6g,h where lengths and diameters are indicated as expanded and after rescaling.

#### Length of the motile cilia

Cilia were manually traced using the segmented line tool of ImageJ^42^. The total measured length was divided by the expansion factor of the gel and reported as a dot plot using GraphPad (https://www.graphpad.com/).

#### Area and roundness of the pyrenoids

Methanol-fixed and cryofixed pyrenoids were manually delineated using the polygon selection tool of ImageJ. Roundness and area were calculated and reported as dot plot using GraphPad.

#### GFP-Sec61β intensity signal measurement

For the comparison of GFP-Sec61β signal intensity obtained after cryofixation versus PFA/GA fixation, we measured the mean intensity of identical regions of interest (200 × 200 pixels) from nondeconvolved images of each condition. Five regions of interest per cell were quantified and averaged. Dot-plots were generated using GraphPad.

#### Mitochondrial area and TOMM20 density measurement

For comparison of mitochondrial morphology under different fixative conditions (cryofixation, methanol, PFA and PFA/GA), we binarized deconvolved images on the MitoTracker channel and applied a mask to draw the outlines of the mitochondrial network. We then quantified the surface area of the mitochondrial network and expressed it as a percentage of the whole cell surface area. For the quantification of the TOMM20 density under different fixative conditions (cryofixation, methanol, PFA and PFA/GA), we manually counted the number of TOMM20 dots in 3–5 mitochondria per cell and expressed it as dots μm^−2^. The surface of mitochondria was calculated on the MitoTracker channel as above. Dot-plots were generated using GraphPad.

#### Mitotic spindle measurements

RPE-1 cells were cryofixed and either directly stained with α/β-tubulin antibodies and DAPI or processed for U-ExM and post-stained with α/β-tubulin antibodies and DAPI. Quantification of the mitotic spindle length at different mitotic stages (prophase, metaphase and anaphase) was performed with the straight-line tool of image and plotted with GraphPad.

#### Plot profiles

Plot profiles from Fig. 4j, Extended Data Fig. 4f and Supplementary Fig. 3f were obtained using the straight-line tool of ImageJ and plotted using GraphPad. The distance between either half of the maximal (Extended Data Figs. 4f and 6g) or peak-to-peak (Extended Data Fig. 6h) distance was calculated.

#### RMS calculation on U2OS cells expressing LifeAct-GFP

U2OS were cultured, transfected with LifeAct-GFP plasmid and cryofixed as described above. GFP-positive cells in a restricted area at the center of the coverslips were rapidly acquired and the coverslips were directly processed for U-ExM as described above. The center of the gel was stained with anti-GFP (see immunostaining section) and acquired with the same microscope as previous expansion. To estimate the sample deformation after expansion, we calculated the r.m.s. error between two images of the same structure before and after expansion, following the protocol described by Chozinski et al^43^. This protocol also provides the scale factor between the images, thus giving the expansion factor of the experiment.

### Statistical analysis

The comparison of two groups was performed using a two-sided Student’s *t*-test or its nonparametric correspondent, the Mann–Whitney *U*-test, if normality was not granted either because of not being checked (*n* < 10) or because it was rejected (D’Agostino and Pearson test). The comparisons of more than two groups were made using one-way ANOVA followed by post hoc tests (Kruskal–Wallis test) to identify all the significant group differences. *n* indicates independent biological replicates from distinct samples. Data are represented as scatter-plots with center line as mean. The graphs with error bars indicate 1 s.d. (±) and the significance level is denoted as usual (*P < 0.05, **P < 0.01, ***P < 0.001). All statistical analyses were performed using Prism7 (GraphPad v.7.0a).

### Reproducibility

All experiments were performed at least three times, except for the ependymal cells and neurons, which were performed only once. Representative images are shown for each experiment.

### Reporting Summary

Further information on research design is available in the Nature Research Reporting Summary linked to this article.

## Online content

Any methods, additional references, Nature Research reporting summaries, source data, extended data, supplementary information, acknowledgements, peer review information; details of author contributions and competing interests; and statements of data and code availability are available at 10.1038/s41592-021-01356-4.

## Supplementary information

Supplementary Information Supplementary Figs. 1–5 with associated legends.

Reporting Summary

Supplementary Data 1 Statistical source data corresponding to Supplementary Fig. 2f,g.

## Data Availability

The data that support the findings of this study are available as ‘source data’ provided with the manuscript. Further request can be sent to the corresponding authors. Source data are provided with this paper.

## Source data

Source Data Fig. 2 Statistical source data corresponding to Fig. 2j.
Source Data Fig. 4 Statistical source data corresponding to Fig. 4c,g.
Source Data Fig. 5 Statistical source data corresponding to Fig. 5i.
Source Data Extended Data Fig. 6 Statistical source data corresponding to Extended Data Fig. 6c,g,h.
Source Data Extended Data Fig. 7 Statistical source data corresponding to Extended Data Fig. 7b.
Source Data Extended Data Fig. 10 Statistical source data corresponding to Extended Data Fig. 10c.

## References

[1] Chen F, Tillberg PW, Boyden ES (2015). Optical imaging. Expansion microscopy. Science.

[2] Sahl SJ, Hell SW, Jakobs S (2017). Fluorescence nanoscopy in cell biology. Nat. Rev. Mol. Cell Biol..

[3] Schnell U, Dijk F, Sjollema KA, Giepmans BNG (2012). Immunolabeling artifacts and the need for live-cell imaging. Nat. Methods.

[4] Dubochet J (2012). Cryo-EM-the first thirty years. J. Microsc..

[5] Neuhaus EM, Horstmann H, Almers W, Maniak M, Soldati T (1998). Ethane-freezing/methanol-fixation of cell monolayers: a procedure for improved preservation of structure and antigenicity for light and electron microscopies. J. Struct. Biol..

[6] Phillips MA (2020). CryoSIM: super-resolution 3D structured illumination cryogenic fluorescence microscopy for correlated ultrastructural imaging. Optica.

[7] Kounatidis I (2020). 3D correlative cryo-structured illumination fluorescence and soft X-ray microscopy elucidates reovirus intracellular release pathway. Cell.

[8] Hoffman DP (2020). Correlative three-dimensional super-resolution and block-face electron microscopy of whole vitreously frozen cells. Science.

[9] Moser F (2019). Cryo-SOFI enabling low-dose super-resolution correlative light and electron cryo-microscopy. Proc. Natl Acad. Sci. USA.

[10] Gambarotto D (2019). Imaging cellular ultrastructures using expansion microscopy (U-ExM). Nat. Methods.

[11] Gambarotto, D., Hamel, V. & Guichard, P. in *Methods in Cell Biology* Ch. 4 10.1016/bs.mcb.2020.05.006 (2021). 10.1016/bs.mcb.2020.05.006 33478697

[12] Kellenberger E (1991). The potential of cryofixation and freeze substitution: observations and theoretical considerations. J. Microsc..

[13] Mahamid J (2016). Visualizing the molecular sociology at the HeLa cell nuclear periphery. Science.

[14] Dubochet J, Sartori Blanc N (2001). The cell in absence of aggregation artifacts. Micron.

[15] Guo Y (2018). Visualizing intracellular organelle and cytoskeletal interactions at nanoscale resolution on millisecond timescales. Cell.

[16] Malhas A, Goulbourne C, Vaux DJ (2011). The nucleoplasmic reticulum: form and function. Trends Cell Biol..

[17] Nixon-Abell J (2016). Increased spatiotemporal resolution reveals highly dynamic dense tubular matrices in the peripheral ER. Science.

[18] M’Saad O, Bewersdorf J (2020). Light microscopy of proteins in their ultrastructural context. Nat. Commun..

[19] Mao C (2020). Feature-rich covalent stains for super-resolution and cleared tissue fluorescence microscopy. Sci. Adv..

[20] Leterrier C (2021). A pictorial history of the neuronal cytoskeleton. J. Neurosci..

[21] Blanchoin L, Boujemaa-Paterski R, Sykes C, Plastino J (2014). Actin dynamics, architecture, and mechanics in cell motility. Physiol. Rev..

[22] Riedl J (2008). Lifeact: a versatile marker to visualize F-actin. Nat. Methods.

[23] Ponjavić, I., Vukušić, K. & Tolić, I. M. in *Methods in Cell Biology* Ch. 12 10.1016/bs.mcb.2020.04.014 (2021). 10.1016/bs.mcb.2020.04.014 33478692

[24] Bosch Grau M (2013). Tubulin glycylases and glutamylases have distinct functions in stabilization and motility of ependymal cilia. J. Cell Biol..

[25] Nguyen QPH (2020). Comparative super-resolution mapping of basal feet reveals a modular but distinct architecture in primary and motile cilia. Dev. Cell.

[26] Teves SS (2016). A dynamic mode of mitotic bookmarking by transcription factors. eLife.

[27] Stanly TA (2016). Critical importance of appropriate fixation conditions for faithful imaging of receptor microclusters. Biol. Open.

[28] AbuZineh K (2018). Microfluidics-based super-resolution microscopy enables nanoscopic characterization of blood stem cell rolling. Sci. Adv..

[29] Freeman Rosenzweig ES (2017). The eukaryotic CO_2_-concentrating organelle is liquid-like and exhibits dynamic reorganization. Cell.

[30] Elbaum-Garfinkle S (2015). The disordered P granule protein LAF-1 drives phase separation into droplets with tunable viscosity and dynamics. Proc. Natl Acad. Sci. USA.

[31] Halpern AR, Alas GCM, Chozinski TJ, Paredez AR, Vaughan JC (2017). Hybrid structured illumination expansion microscopy reveals microbial cytoskeleton organization. ACS Nano..

[32] Gao M (2018). Expansion stimulated emission depletion microscopy (ExSTED). ACS Nano..

[33] Zwettler FU (2020). Molecular resolution imaging by post-labeling expansion single-molecule localization microscopy (Ex-SMLM). Nat. Commun..

[34] Hamel V (2017). Identification of chlamydomonas central core centriolar proteins reveals a role for human WDR90 in ciliogenesis. Curr. Biol..

[35] Chassefeyre R (2015). Regulation of postsynaptic function by the dementia-related ESCRT-III subunit CHMP2B. J. Neurosci..

[36] Mercey O (2019). Dynamics of centriole amplification in centrosome-depleted brain multiciliated progenitors. Sci. Rep..

[37] Zhu Y (2018). Sec61β facilitates the maintenance of endoplasmic reticulum homeostasis by associating microtubules. Protein Cell.

[38] Tivol WF, Briegel A, Jensen GJ (2008). An improved cryogen for plunge freezing. Microsc. Microanal..

[39] Le Guennec M (2020). A helical inner scaffold provides a structural basis for centriole cohesion. Sci. Adv..

[40] ALAMOUDI A, STUDER D, DUBOCHET J (2005). Cutting artefacts and cutting process in vitreous sections for cryo-electron microscopy. J. Struct. Biol..

[41] Nizak C (2003). Recombinant antibodies against subcellular fractions used to track endogenous Golgi protein dynamics in vivo. Traffic.

[42] Schneider CA, Rasband WS, Eliceiri KW (2012). NIH Image to ImageJ: 25 years of image analysis. Nat. Methods.

[43] Chozinski TJ (2016). Expansion microscopy with conventional antibodies and fluorescent proteins. Nat. Methods.

