# Supplementary material for: Visualizing the native cellular organization by coupling cryofixation with expansion microscopy (Cryo-ExM)
Source: Nat Methods. 2022 Jan 13;19(2):216–22. doi: 10.1038/s41592-021-01356-4 (PMC8828483; doi:10.1038/s41592-021-01356-4)
Supplement: Supplementary file 1 — Supplementary Figs. 1–5 with associated legends. [file 41592_2021_1356_MOESM1_ESM.pdf]

nature portfolio

https://doi.org/10.1038/s41592-021-01356-4

Supplementary information

Visualizing the native cellular organization by coupling cryofixation with expansion microscopy (Cryo-ExM)

In the format provided by the authors and unedited

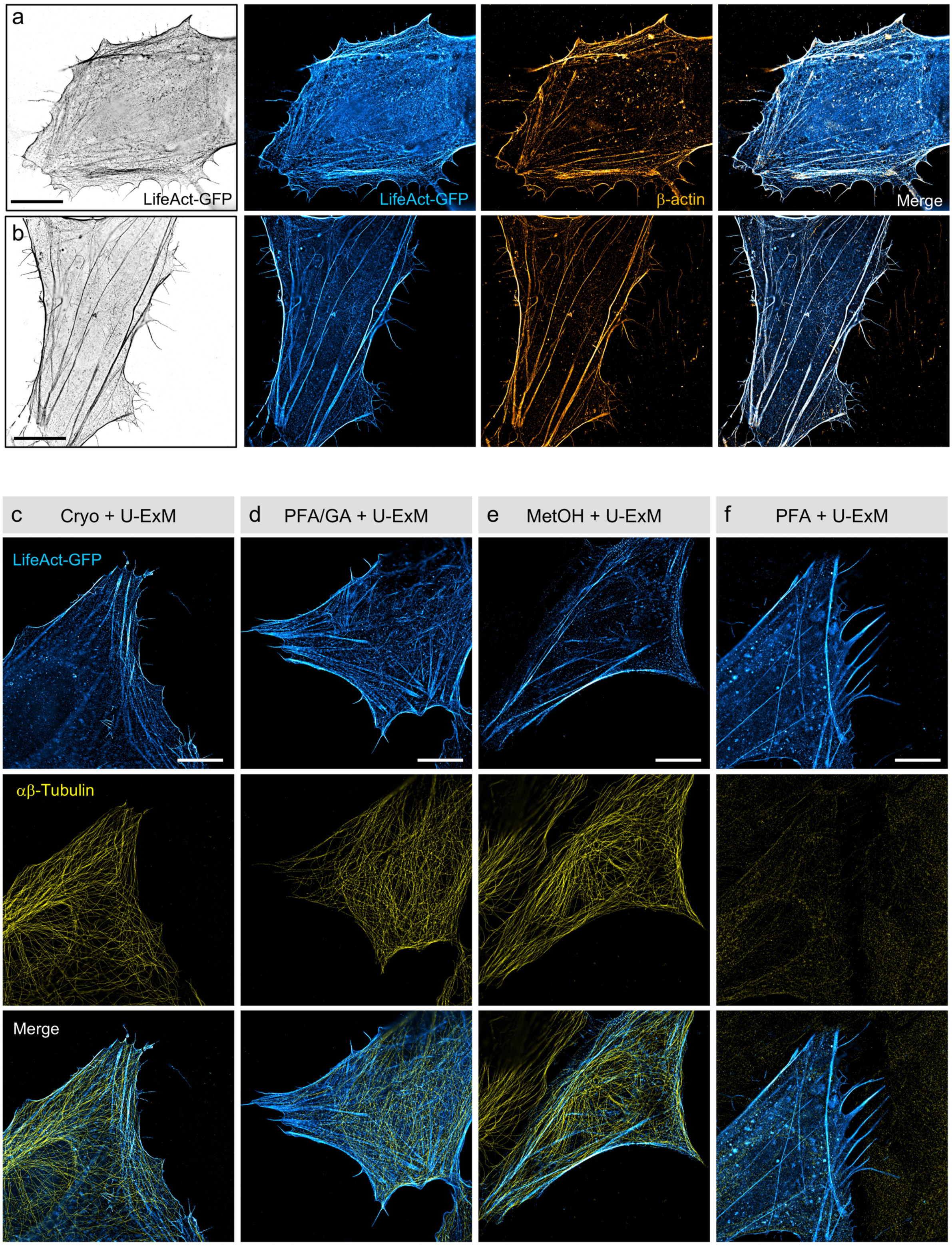

Supplementary Figure 1. Effect of fixation on actin network. (**a**, **b**) Widefield images of expanded cryo-fixed U2OS cell expressing LifeAct-GFP stained for GFP (grey/ cyan) and β-actin (orange hot) showing the overlapping signal of both probes. Scale bar: 10 µm. (**c-f**) Widefield images of expanded U2OS expressing LifeAct-GFP stained for GFP (cyan) and α/β-Tubulin (yellow) after cryo (c), PFA/GA (d), MetOH (e) or PFA (f) fixation. Scale bar: 10 µm.

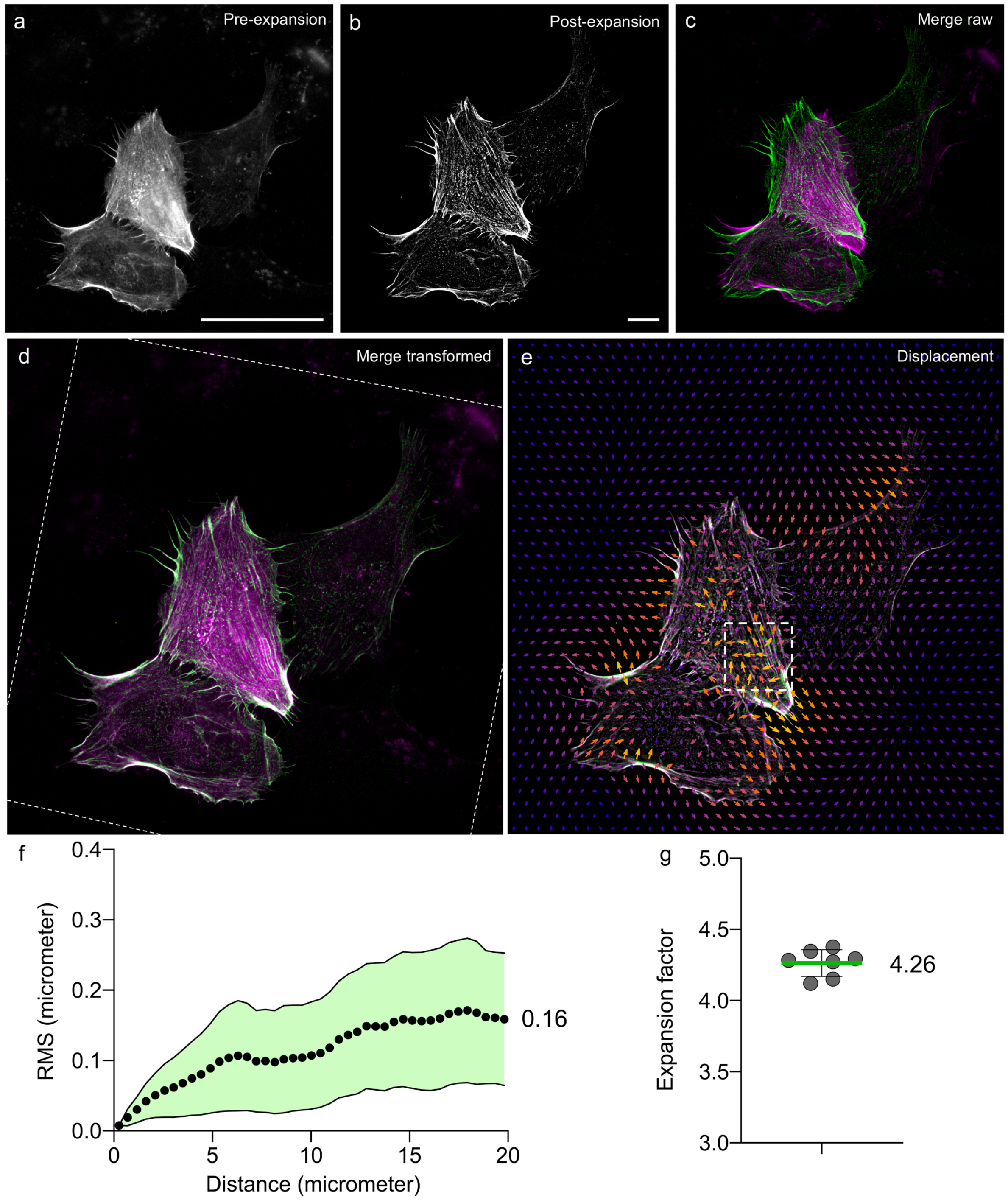

Supplementary Figure 2. Distortion calculation for LifeAct-GFP after cryo-ExM. (a, b) Widefield images of a U2OS cell expressing LifeAct-GFP acquired pre- (**a**) and post-expansion (**b**). Scale bar = 50 µm. (**c**) Overlay of pre- and post-expansion images shown in (**a**, **b**). (**d**) Overlay of pre-expansion image (magenta) and post-expansion image (green) after alignment of the post-expansion image using similarity registration (i.e., translation, rotation, and magnification—see methods for further details). **(e)** Overlay of post-expansion images before (magenta) and after (green) a non-rigid transformation procedure that uses B-spline registration to deform the post-expansion image, in order to optimally fit the pre-expansion image. Arrows

indicate the direction and relative magnitude (purple to yellow) of the transformation required to optimally align the post-expansion to the pre-expansion image. (**f**) Quantification of root mean square (RMS) error over distance (micrometers) comparing pre- (**a**) and post- (**b**) expansion. Line connects the mean of each x value and error bars represent the standard deviation. (**g**) Calculated expansion factor based on the registration used to compare pre- and post- expansion images (a-c). Average +/- SD: 4.26 +/- 0.094. N = 7 cells from 1 experiment.

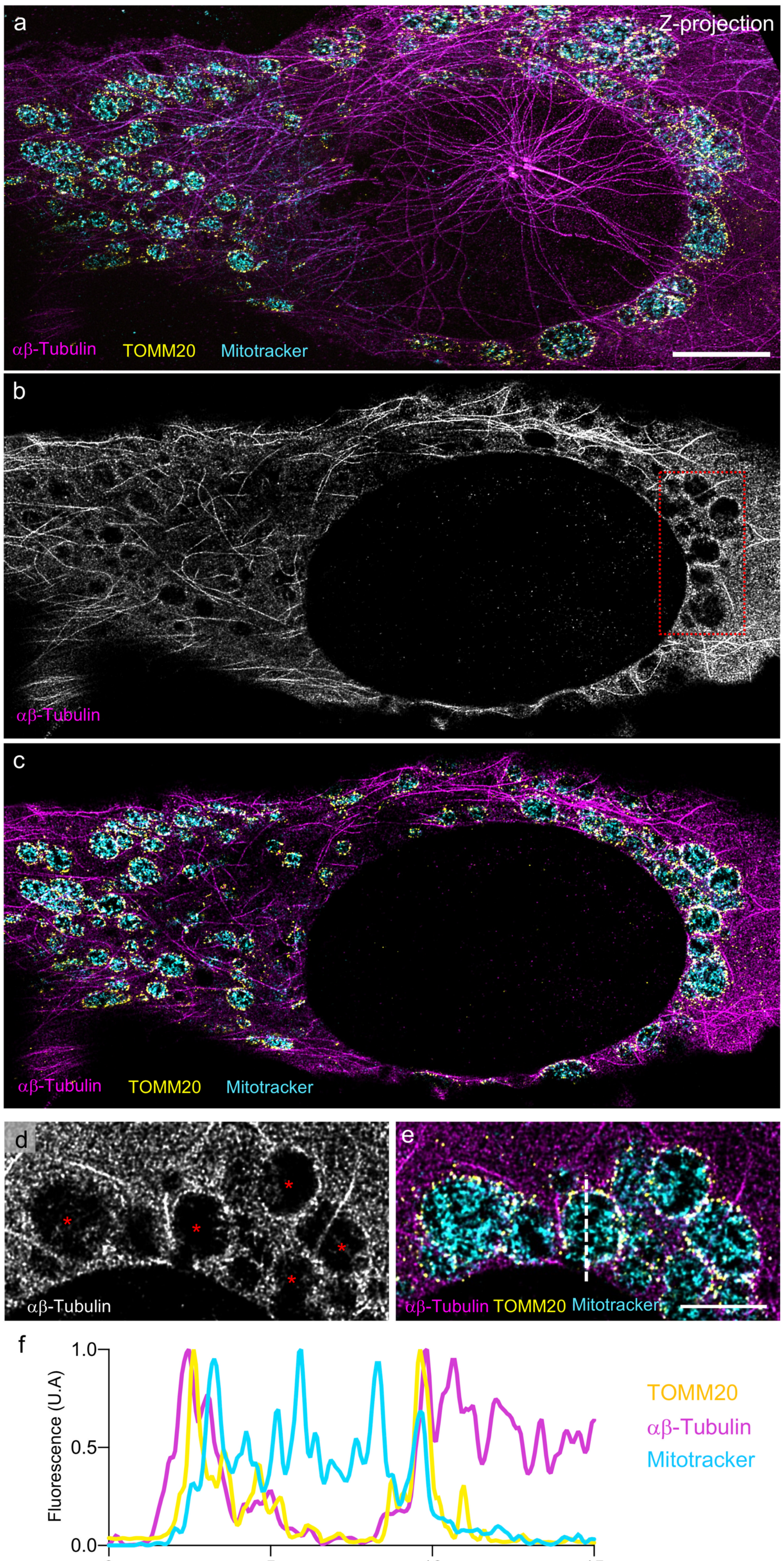
a
Z-projection
αβ-Tubulin
TOMM20
Mitotracker
b
αβ-Tubulin
c
αβ-Tubulin
TOMM20
Mitotracker
d
αβ-Tubulin
e
αβ-Tubulin TOMM20 Mitotracker
f
Fluorescence (U.A)
1.0
0.5
0.0
0
5
10
15
TOMM20
αβ-Tubulin
Mitotracker

Supplementary Figure 3. Cytoplasmic pool of tubulin revealed by cryo-ExM. (**a-c**) Confocal image of cryo-fixed cell, expanded and stained with α/β-tubulin (magenta), TOMM20 (yellow) and mitotracker (cyan) to visualize mitochondrial outer membrane and matrix respectively (**a**, z-projection; **b**, **c**: single plan). Red dashed square indicates the inset shown in **d**, **e**. (**d**, **e**) Single/double channel images showing the absence of tubulin (**d**, red stars) at the position of the mitochondria labeled with TOMM20 and mitotracker (**e**). Scale bars= 5 μm (**a**) and 2 μm (**d**). (**f**) Plot profile across a single mitochondrion (dashed line from (**e**)) showing the exclusive localization of tubulin (α/β-tubulin) and mitotracker / TOMM20 delineating the mitochondrion.

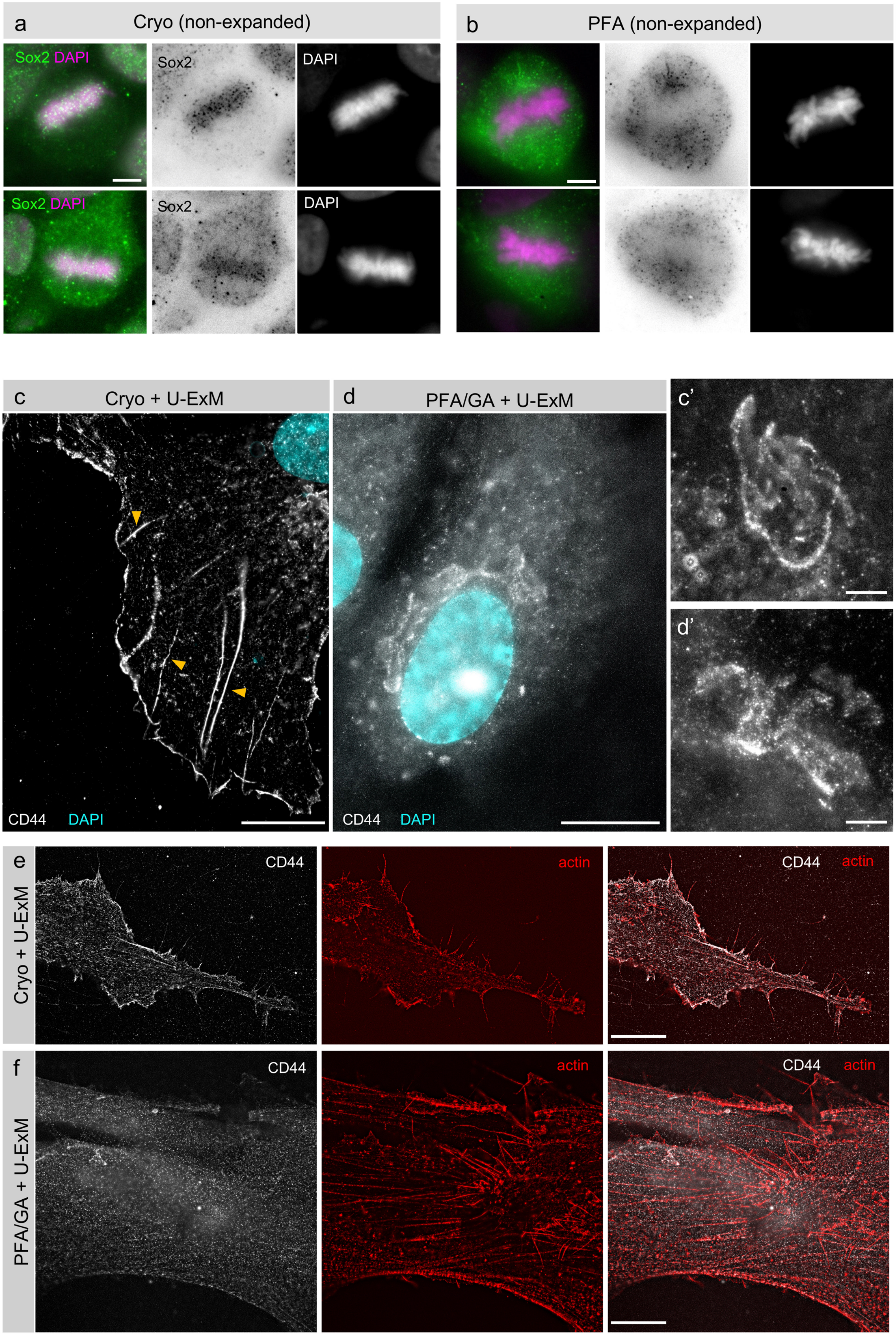
a
Cryo (non-expanded)
Sox2 DAPI
Sox2
DAPI
b
PFA (non-expanded)
c
Cryo + U-ExM
d
PFA/GA + U-ExM
c'
d'
CD44 DAPI
e
Cryo + U-ExM
CD44
actin
f
PFA/GA + U-ExM

Supplementary Figure 4. Effect of fixation on the transcription factor Sox2 and the cell surface glycoprotein CD44. (**a**, **b**) Widefield images of non-expanded HEK cells undergoing mitosis, stained for the transcription factor Sox2 (grey, green) and DAPI (magenta) after cryo-fixation (**a**) or PFA fixation (**b**). Note that the chromosomal retention of Sox2 during mitosis is only observable after cryo-fixation. Scale bar= 5 µm. (**c**, **d**) Widefield images of expanded RPE-1 cells stained for CD44 (grey) and DAPI (cyan) after cryo-fixation (**c**) or PFA/GA (**d**). Yellow arrowheads indicate CD44 positive fibers observed only in the cryo-fixed cells but not after PFA/GA fixation. Scale bar= 10 µm. (**c'**, **d'**) Localization of CD44 at the level of golgi in both cryo-fixed (**c'**) and PFA/GA-fixed cells (**d'**). Scale bar= 2µm. (**e**, **f**) Widefield images of expanded RPE-1 cells stained for CD44 (grey) and actin (red) after cryo-fixation (**e**) or PFA/GA (**f**). Note that the colocalization between CD44 and actin could only be observed in cryo-fixed cells. Scale bar= 10 µm.

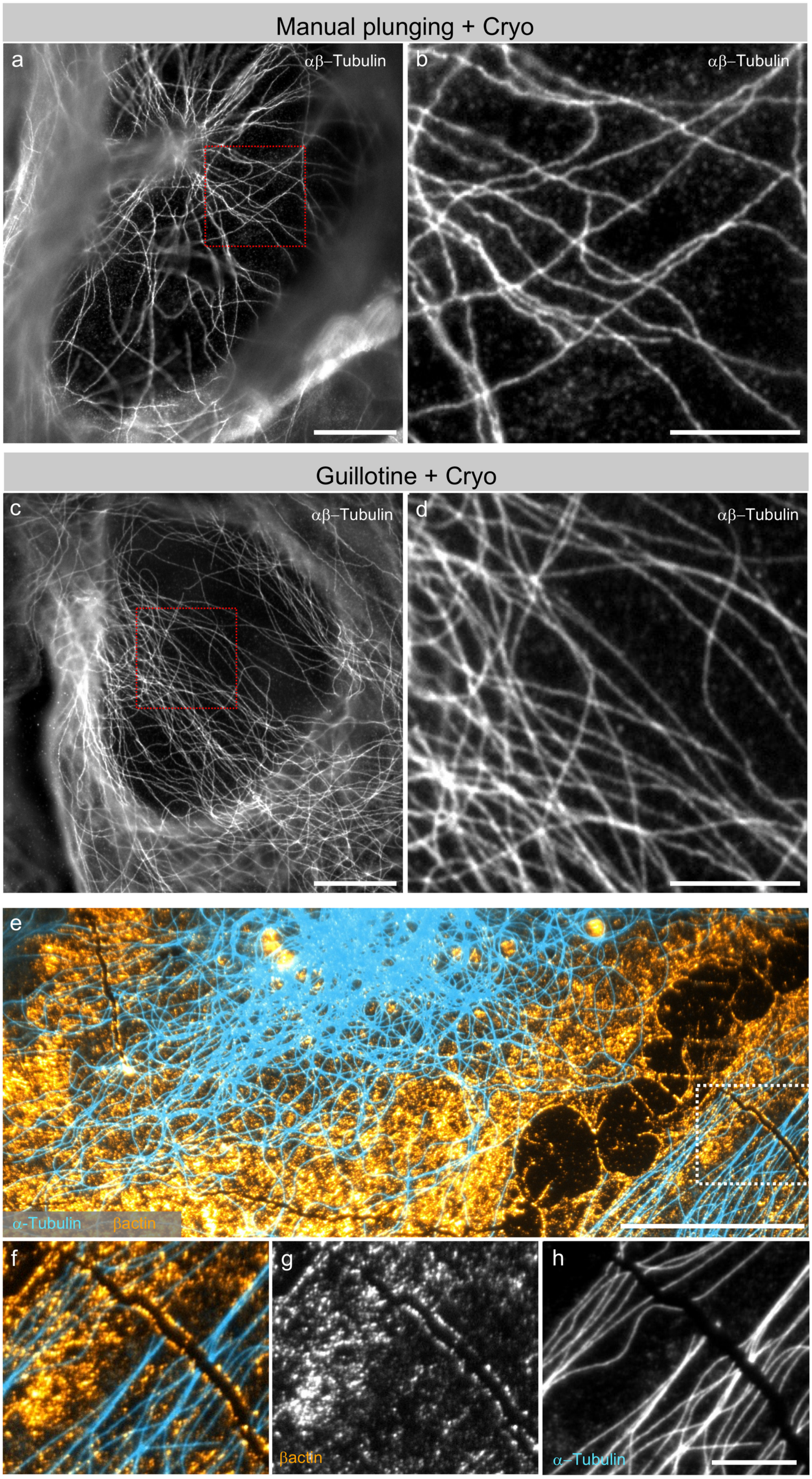

Manual plunging + Cryo
a
αβ–Tubulin
b
αβ–Tubulin
Guillotine + Cryo
c
αβ–Tubulin
d
αβ–Tubulin
e
α-Tubulin
βactin
f
g
βactin
h
α–Tubulin

Supplementary Figure 5. Plunging speed effects in Cryo-ExM (**a**, **b**) Widefield images of expanded U2OS cryo-fixed by manual plunging, stained for α/β-tubulin. Inset depicted by red square shows the waviness of the microtubules due to the low-speed plunging into liquid ethane (**b**). Scale bars = 5 µm (**a**), 2 µm (**b**). (**c**, **d**) Widefield images of expanded U2OS cryo-fixed by guillotine plunging stained for α/β-tubulin. Inset depicted by red square shows the intact microtubule network preserved due to the high-speed plunging into liquid ethane (**d**). Scale bars = 5 µm (**c**), 2 µm (**d**). (**e**-**h**) Widefield image of cryo-fixed, expanded U2OS cell stained with α-tubulin (cyan) and β-actin (orange hot) showing fractures due to the freezing procedure, a frequently observed feature in cryo-electron microscopy. Insets show a closer look on one fracture. Scale bars= 10 µm (**d**), 2 µm (**h**).
