# Supplementary material for: Visualizing the native cellular organization by coupling cryofixation with expansion microscopy (Cryo-ExM)
Source: Nat Methods. 2022 Jan 13;19(2):216–22. doi: 10.1038/s41592-021-01356-4 (PMC8828483; doi:10.1038/s41592-021-01356-4)
Supplement: Supplementary file 2 — Reporting Summary [file 41592_2021_1356_MOESM2_ESM.pdf]

nature research

Corresponding author(s): Paul Guichard

Last updated by author(s): 29/10/2021

# Reporting Summary

Nature Research wishes to improve the reproducibility of the work that we publish. This form provides structure for consistency and transparency in reporting. For further information on Nature Research policies, see our Editorial Policies and the Editorial Policy Checklist.

## Statistics

For all statistical analyses, confirm that the following items are present in the figure legend, table legend, main text, or Methods section.

| n/a | Confirmed | |
|---|---|---|
| ☐ | ☒ | The exact sample size (*n*) for each experimental group/condition, given as a discrete number and unit of measurement |
| ☐ | ☒ | A statement on whether measurements were taken from distinct samples or whether the same sample was measured repeatedly |
| ☐ | ☒ | The statistical test(s) used AND whether they are one- or two-sided<br>*Only common tests should be described solely by name; describe more complex techniques in the Methods section.* |
| ☒ | ☐ | A description of all covariates tested |
| ☒ | ☐ | A description of any assumptions or corrections, such as tests of normality and adjustment for multiple comparisons |
| ☐ | ☒ | A full description of the statistical parameters including central tendency (e.g. means) or other basic estimates (e.g. regression coefficient) AND variation (e.g. standard deviation) or associated estimates of uncertainty (e.g. confidence intervals) |
| ☒ | ☐ | For null hypothesis testing, the test statistic (e.g. *F*, *t*, *r*) with confidence intervals, effect sizes, degrees of freedom and *P* value noted<br>*Give P values as exact values whenever suitable.* |
| ☒ | ☐ | For Bayesian analysis, information on the choice of priors and Markov chain Monte Carlo settings |
| ☒ | ☐ | For hierarchical and complex designs, identification of the appropriate level for tests and full reporting of outcomes |
| ☒ | ☐ | Estimates of effect sizes (e.g. Cohen's *d*, Pearson's *r*), indicating how they were calculated |

*Our web collection on statistics for biologists contains articles on many of the points above.*

## Software and code

Policy information about availability of computer code

| | |
|---|---|
| Data collection | Image acquisition was performed on an inverted Leica TCS SP8 microscope or on a Leica Thunder DMi8 microscope using a 63x 1.4 NA oil objective with Lightening or Thunder SVCC (small volume computational clearing) mode at max resolution, adaptive as 'Strategy' and water as 'Mounting medium' to generate deconvolved images. 3D stacks were acquired with 120nm z-intervals and an x, y pixel size of 35 nm (Leica TCS SP8) or 210nm z-intervals and an x, y pixel size of 100 nm (Thunder DMi8). |
| Data analysis | The images were generated with the software ImageJ (version 2.0.0-rc-69/1.53c) and graphpad was used for graphical representation and statistics (version 7.0a, April 2, 2016) |

For manuscripts utilizing custom algorithms or software that are central to the research but not yet described in published literature, software must be made available to editors and reviewers. We strongly encourage code deposition in a community repository (e.g. GitHub). See the Nature Research guidelines for submitting code & software for further information.

## Data

Policy information about availability of data

All manuscripts must include a data availability statement. This statement should provide the following information, where applicable:

- Accession codes, unique identifiers, or web links for publicly available datasets
- A list of figures that have associated raw data
- A description of any restrictions on data availability

The data that support the findings of this study are available as 'source data' provided with the manuscript . Further request can be sent to the corresponding authors.

# Field-specific reporting

Please select the one below that is the best fit for your research. If you are not sure, read the appropriate sections before making your selection.

☒ Life sciences ☐ Behavioural & social sciences ☐ Ecological, evolutionary & environmental sciences

For a reference copy of the document with all sections, see nature.com/documents/nr-reporting-summary-flat.pdf

# Life sciences study design

All studies must disclose on these points even when the disclosure is negative.

| | |
|---|---|
| Sample size | We did not use a predetermined sample size. Each staining was repeated 3 times from independent cultures with the exception of the primary culture which was only performed once. For primary culture of eppendymal and neuronal cells, each staining was still repeated a minimum of 3 times from different wells. All numbers used for quantification is specified in the figure legends. |
| Data exclusions | no exclusion was done. |
| Replication | All experiments were performed at least 3 times except for the ependymal cells and neurons, which were performed only once as written in the manuscript. All the attempts were successful. |
| Randomization | This is not relevant for our study because we selected representative regions for every experiements |
| Blinding | Blinding is not relevant for our study for the same reasons as specified above. |

# Reporting for specific materials, systems and methods

We require information from authors about some types of materials, experimental systems and methods used in many studies. Here, indicate whether each material, system or method listed is relevant to your study. If you are not sure if a list item applies to your research, read the appropriate section before selecting a response.

## Materials & experimental systems

| n/a | Involved in the study | |
|---|---|---|
| ☐ | ☒ | Antibodies |
| ☐ | ☒ | Eukaryotic cell lines |
| ☒ | ☐ | Palaeontology and archaeology |
| ☐ | ☒ | Animals and other organisms |
| ☒ | ☐ | Human research participants |
| ☒ | ☐ | Clinical data |
| ☒ | ☐ | Dual use research of concern |

## Methods

| n/a | Involved in the study | |
|---|---|---|
| ☒ | ☐ | ChIP-seq |
| ☒ | ☐ | Flow cytometry |
| ☒ | ☐ | MRI-based neuroimaging |

## Antibodies

Antibodies used

- tubulin monobodies AA344 and AA345
- mouse monoclonal anti beta-actin antibody (Clone: 7D2C10, 1/250, Proteintech 60008-1-1)
- rabbit polyclonal anti-α-tubulin (1:250, ab18251, Abcam)
- rabbit polyclonal anti-PolyE (1:500, AG-25B-0030, AdipoGen)
- rabbit polyclonal anti-GFP (1/250, TP401, Torrey pines)
- rabbit polyclonal anti-TOMM20 (1/250, ab186734, Abcam)
-rabbit polyclonal anti-GM130 (1/250, 11308-1-AP, Proteintech)
-rabbit polyclonal anti-actin (1/250, ab1801, Abcam)
-rabbit polyclonal anti-NUP205 (1/250, 24439-1-AP, Proteintech)
-rabbit polyclonal anti-LC3 (1/250, 14600-1-AP, Proteintech)
-rabbit polyclonal anti-Lamp1 (1/250, D2D11, cell Signaling)
-rabbit polyclonal anti-Sox2 (1/250, 20118-1-AP,Proteintech)
-mouse monoclonal anti-CD44 (clone :6F4H2, 1/250, 60224-1-Ig, Proteintech)

The following secondary antibodies were used: goat anti-rabbit Alexa Fluor 488 IgG H+L (1:400, A11008) and goat anti-mouse Alexa Fluor 568 IgG H+L (1:250, A11004) (Invitrogen, ThermoFisher). This information can be found in the online methods.

Validation

- Validation AA344 : https://oap.unige.ch/journals/abrep/article/view/108
- Validation AA345 : https://oap.unige.ch/journals/abrep/article/view/260 and https://oap.unige.ch/journals/abrep/article/view/108
- Validation 60008-1-1 : western blot analysis: https://www.ptglab.com/products/ACTB-Antibody-60008-1-Ig.htm
- Validation ab18251: western blot analysis: https://www.abcam.com/alpha-tubulin-antibody-microtubule-marker-ab18251.html

- Validation AG-25B-0030: this antibody recognizes specifically glutamate chains of four or more glutamates. https://adipogen.com/ag-25b-0030-anti-polyglutamate-chain-polye-pab-in105.html/
- Validation TP401: western blot analysis: https://www.amsbio.com/rabbit-anti-gfp-pab-tp401
- Validation ab186734: western blot analysis https://www.abcam.com/tomm20-antibody-epr15581-39-mitochondrial-marker-ab186734.html
- Validation 11308-1-AP: western blot analysis https://www.ptglab.com/products/GOLGA2,GM130-Antibody-11308-1-AP.htm
- Validation ab1801 : western blot analysis https://www.abcam.com/actin-antibody-loading-control-ab1801.html
-Validation 24439-1-AP : western blot analysis https://www.ptglab.com/products/NUP205-Antibody-24439-1-AP.htm
- Validation 14600-1-AP : western blot analysis https://www.ptglab.com/products/MAP1LC3B-Antibody-14600-1-AP.htm
- Validation D2D11 : western blot analysis https://www.cellsignal.com/products/primary-antibodies/lamp1-d2d11-xp-rabbit-mab/9091
-Validation 20118-1-AP : western blot analysis https://www.ptglab.com/products/SOX2-Antibody-20118-1-AP.htm
-Validation 60224-1-Ig : western blot analysis https://www.ptglab.com/products/CD44-Antibody-60224-1-Ig.htm

# Eukaryotic cell lines

Policy information about cell lines

| | |
|---|---|
| Cell line source(s) | U2OS, gift from the Nigg laboratory (unknown supplier). RPE-1, gift from the Paoletti laboratory (ATCC) . MEF, gift from the Sadoul laboratory where they were generated . HEK, gift from Martinou laboratory (unknown supplier). |
| Authentication | U2OS cell line was authenticated by profiling using highly polymorphic short tandem repeat loci (STR) (Microsynth). The other cell lines were not authenticated |
| Mycoplasma contamination | Cells were regularly tested for mycoplasma contamination and were not positivie for mycoplasma. |
| Commonly misidentified lines (See ICLAC register) | No commonly misidentified cell lines were used in this study. |

# Animals and other organisms

Policy information about studies involving animals; ARRIVE guidelines recommended for reporting animal research

| | |
|---|---|
| Laboratory animals | C57BL/6J mouse E18.5 embryos and new born (Post-natal day 1) were used to generate mouse neuronal cell culture and eppendymal cell culture respectively. Note that the sex was not determined. |
| Wild animals | No wild animals were used in this study |
| Field-collected samples | No field-collected samples were used in this study. |
| Ethics oversight | The pups were provided for the neuronal culture by the Martinou laboratory (Geneva, Switzerland) under the authorization number GE/205/17. All ethics relative to work with animals were fully respected and experiments were carried out in accordance with the Institutional Animal Care and Use Committee of the University of Geneva and with permission of the Geneva cantonal authorities. |

Note that full information on the approval of the study protocol must also be provided in the manuscript.
